# Augmented wealth in Switzerland: the influence of pension wealth on wealth inequality

**DOI:** 10.1186/s41937-020-00063-9

**Published:** 2020-11-05

**Authors:** Ursina Kuhn

**Affiliations:** Swiss Centre of Expertise in the Social Sciences (FORS), C/o Université de Lausanne, Bâtiment Géopolis, 1015 Lausanne, Switzerland

**Keywords:** Net worth, Augmented wealth, Pension wealth, Wealth inequality, Life cycle, SILC, Data linkage

## Abstract

Entitlements for social security and occupational pensions present a major wealth component and play a central role for financial security. However, most individual-level data lacks information on pension wealth. By linking various data sources, this contribution estimates the present value of future pension entitlements in Switzerland for statutory pensions, occupational pensions and third pillar accounts and analyses the distribution of augmented wealth, which combines pension wealth and net worth. The CH-SILC survey from 2015 is used to estimate real assets, financial assets and pension wealth of retired individuals. The pension entitlements of non-retired individuals are simulated on the basis of their earning history from administrative records following the accrual method and assuming a real discount rate of 2%. When pension wealth is added to net worth, average wealth doubles, and the Gini-coefficient declines by 26%. The equalising effect is particularly strong for social security pensions. The wealth distribution differs strongly between the three pillars of the pension system; there are also strong differences between gender and age groups. In Switzerland, wealth accumulation continues after retirement age.

## Introduction

The high and rising wealth inequality receives growing attention in modern societies and research on economic inequality. Different surveys (e.g. the Household Finance and Consumption Surveys HFCS, the Survey on Aging and Living Conditions SHARE, or the Luxemburg Wealth Study) have started to collect microdata on wealth. However, most wealth estimates neglect pension wealth that—for most individuals—presents the largest asset.

There are many reasons to consider pension entitlements in wealth analysis. First, the inclusion of pension wealth has profound consequences on the wealth distribution. Second, pension rights are important for the comparison of wealth between countries. According to the standard life-cycle hypothesis, expected pension benefits and private wealth are substitutes (Feldstein, 1974). If pension wealth crowds out private saving, countries with more generous pension schemes should have lower private savings than countries with more limited pension schemes. Third, the omission of pension wealth might result in misleading comparison of wealth between covered and non-covered groups within a country (e.g. wealth differences between employed and self-employed). Fourth, pension wealth has been shown to influence many behaviours and decisions, such as private savings, composition of wealth portfolios or retirement timing. When ignoring pension wealth, studies on these behaviours could yield biased results. Finally, a better knowledge on the inequality of pension wealth can be policy relevant.

At the same time, pension entitlements differ from other assets in several respects: they cannot alleviate income poverty, be used to purchase consumer goods, or be passed on to others. Accordingly, pension entitlements do not confer *political power*, *social influence*, or *social status*. Furthermore, individuals have little control over how their pension assets are invested and restrictions often apply for bequests. Finally, the tax system and welfare state treat pension wealth differently from other assets. A further difference occurs for pay-as-you go systems, where entitlements are not funded.

There is no agreement on how to deal with pension wealth in analysis on wealth inequality. Some scholars argue for ignoring pension wealth, as it does not give “direct personal control over resources” (Alvaredo, Atkinson and Morelli, 2018). The most widely used wealth definition based on microdata is net worth, which refers to the “value of all the assets owned by a household less the value of all its liabilities at a particular point in time” (OECD, 2013, p. 54). Net worth includes private pensions, but not occupational and statutory ones. The term “augmented wealth” refers to the broader definition of wealth that includes entitlements to future pension streams (Davies and Shorrocks, 2000). Organisations such as the OECD suggest using augmented wealth as a measure complementary to net worth (OECD, 2013, p. 67ff). While these definitions are standards in the micro-approach to wealth analyses (see e.g. Wolff, 2015; Cowell, Nolan, Olivera and Van Kerm, 2017, p. 177), they differ from the macro-economic definition of wealth in the core tables in the System of National Accounts (SNA), which includes only funded pension wealth. However, unfunded pension wealth is included in supplementary data of the SNA to facilitate country comparisons (System of National Accounts, 2008, p. 369ff.). Attempts at distributional national accounts, such as the World Inequality Database (WID), usually follow the wealth definition in the core tables of the SNA, which include only funded pension wealth. Apart from theoretical considerations, data availability and data comparability often dictate the wealth definition used in empirical studies.

This study documents the relevance and the distribution of pension wealth in Switzerland and shows how it affects wealth inequality. The analysis is based on a new linkage of administrative and survey data with a matching rate of over 99% of the sample. Survey data come from the CH-SILC in 2015, which contains separate questions on various real and financial assets including private pension accounts. Administrative records are used to estimate entitlements for social security and occupational pensions for non-retired individuals at the basis of yearly incomes since 1981. While the linked data allows a rather precise estimation of statutory pension wealth, the simulation for occupational pension wealth relies on many assumptions. Following the OECD guidelines for Micro Statistics on Household Wealth (OECD, 2013), I apply the accrual method to estimate the present values of pension wealth under the current pension system. For social security pensions and ongoing second-pillar pensions, I estimate the future pension stream on cohort- and gender-specific survival rates assuming a real discount rate of 2%. For the occupational pension, I estimate the accumulated capital, which is influenced neither by life expectancy nor by retirement age.

Several important findings come out of my analysis. In line with experiences from other countries, pension wealth is highly relevant and has an equalising effect on wealth inequality. Twenty-eight percent of augmented wealth consists of entitlements for social security pensions, and another 23% consists of entitlements for occupational pensions. Although the Gini coefficient of net worth amounts to 0.76, it decreases to 0.65 when adding occupational pensions and to 0.55 for augmented wealth. Moreover, there are important wealth differences between men and women and between age groups. In contrast to findings of other countries, wealth accumulation over the life span continues beyond retirement age.

The methodological approach followed has the advantage of making results comparable to estimates for Germany and the USA by Bönke, Grabka, Schröder and Wolff (2019) but comes with some limitations. Firstly, it ignores the heterogeneity in life expectancy by socio-economic groups, which applies also in Switzerland (e.g. Mackenbach et al., 2019; Wanner and Lerch, 2012). As high-income groups live longer compared to low-income groups, they will receive benefits for a longer time and limit the equalising effect of pensions (Haan, Kemptner and Lüthen, 2019). Secondly, the accrual method does not take account of future pension reforms but assesses the situation in 2015. Due to both effects, the redistributive effect of pension wealth will be weaker in reality. The impact of increasing pension age on wealth inequality is simulated as a sensitivity analysis, which shows a small desequalising effect of such reforms.

While analysis of augmented wealth is common in the USA and UK, there have been only a few attempts to measure the distribution of augmented wealth in Europe (Roine and Waldenström, 2009 for Sweden; Maunu, 2010 for Finland; Bönke, Grabka, Schröder, Wolff and Zyska, 2019 for Germany). All studies reveal a mitigating effect of pension assets on wealth inequality. To my knowledge, there have been no attempts so far to empirically measure augmented wealth in Switzerland. This country presents an interesting case, as it has a very high wealth concentration at the top but a rather average wealth inequality when measured with the Gini index. Moreover, the share of private occupational pensions is (with 31% of post-retirement disposable income) among the highest in OECD countries (OECD, 2019).

A simulation for top wealth shares was conducted by Föllmi and Martínez (2017) based on two simplified scenarios for occupational and private pension wealth but not social security pensions. Assuming an equal distribution of pension assets, the simulation showed that the wealth share of the top 10% of the population would decrease by 27 percentage points. Assuming that shares in pension wealth would correspond to share in labour income, the top 10% wealth share was lowered by 20 percentage points. The individual data used in my study show a slightly weaker reduction in inequality because occupational pensions are more unequally distributed than labour income and because pension wealth and other assets are positively correlated.

A related body of literature addresses the effect of pension entitlements on savings. According to the life cycle hypothesis, individuals save during their working lives and commence with dissaving after retirement (Modigliani, 1986). Accordingly, a change in the expected pension benefits should alter private wealth by the same amount (Feldstein, 1974). Although empirical studies find evidence for such crowding out, the rate is considerably lower than one (e.g. Attanasio and Brugiavini, 2003; Bottazzi, Jappelli and Padula, 2006; Chetty, Friedman, Leth-Petersen, Nielsen and Olsen, 2014; Feng, He and Sato, 2011). Explanations for the limited substitution include bequest motives, short-sightedness, liquidity constraints, risk associated with future reforms, individual information and non-marketable future benefits (Bottazzi et al., 2006, p. 2188).

This paper is structured as follows. In Section 2, I briefly present the pension system in Switzerland and review literature on wealth inequality in Switzerland. Section 3 describes the data and methodological approaches used. Section 4 documents the estimation of pension wealth. Results are discussed in Section 5; Section 6 discusses pension reforms and provides sensitivity analysis for the impact of widowhood pensions, different discount rates and increasing retirement age; and Section 7 concludes.

## Background: the pension system and wealth distribution in Switzerland

**The Swiss pension system** consists of three pillars: the statutory Old Age and Survivors’ Insurance (OASI), occupational pension plans and private voluntary tax-exempt savings. The statutory retirement age is 65 years for men and 64 years for women. In the following, I briefly present each pension type in turn referring to the legislation from 2014. I emphasise restrictions on the use of pension wealth, as these give rise to the arguments for and against including each pension asset in the wealth definition.

The first pillar covers the entire residential population, individuals who migrated to Switzerland after retirement present an exception. The OASI is financed mainly on a pay-as-you-go basis by contributions of 8.4% of the employee’s income without a cap. This rate was increased in 2019 to 8.7%. Childcare years for children under age 16 are additionally credited. Contributions made during marriage are split equally between partners in the event of divorce or once both partners are retired. With full contributions (beginning at the age of 21 and up to the official retirement age), monthly pensions are between 1170 and 2340 CHF (in 2014) depending on the average income during the insurance period. Missing contribution years lead to permanently lowered pensions. Pensions are reduced for early take-up (possible 2 years before regular retirement age) and higher for later retirement. The pension of a married couple is capped at 150% of the maximum individual pension. In case of widowhood, the surviving partner receives a pension. There are no gender differences in pensions: women’s average monthly pension in December 2014 amounted to 2024 CHF, and men’s average pension to 2023 CHF (Federal Social Insurance Office, 2015).

The second pillar of the pension system has a compulsory and supplementary part. Contributions to occupational pensions are compulsory for earnings between CHF 24,570 and CHF 84,240, but all individuals who earn at least 21,060 CHF per year from the same employer are covered with a minimal insured income of 3510 CHF. Therefore, not all part-time workers have a second pillar account. The legislation defines minimum contribution rates that increase with age (from 7% for employees aged 25 to 34 to 18% for employees older than 55 years of age). The pension funds must pay interest on the accumulated capital; a minimum interest rate is stipulated by the Federal Council.

Most pension funds provide benefits in addition to this compulsory insurance. At the end of 2016, there were 1713 pension funds in Switzerland (Swiss Federal Statistical Office, 2017). Most funds also insure income above 84,240 CHF up to the legal threshold of CHF 846,000, or they apply higher contribution rates than the legal minimum. Pension funds are also free to set the interest rate in the voluntary area. Many pension funds apply lower income thresholds for part-time workers. Unfortunately, there are no official statistics on average contribution rates or interest rates.

In addition to fixed contributions from employment income, insured persons can make voluntary payments into the pension fund, which are tax-deductible and therefore most attractive for individuals with a high taxable income. Self-employed individuals can contribute to the second pillar on a voluntary basis.

At retirement, individuals can choose between capital withdrawal and annuitisation of their occupational pension entitlements, but a minimum age of 58 years applies. The choice is influenced by life expectancy, future income, tax rates and individual preferences (Bütler and Ramsden, 2016). At retirement, 50% chose annuities, 32% capital and 19% a combination of capital and yearly pensions (Swiss Federal Statistical Office, 2019b). In the case of annuities, the pension amounts to 6.8% of assets for the mandatory part and can be freely defined by the pension fund for the voluntary part. Because conversion rate of 6.8% is too high considering life expectancy (see e.g. Eling, 2018), the rates are usually much lower in the supplementary part.^1^ In addition, the second pillar can be withdrawn before retirement to acquire a residential property or create a business. The payout of savings is taxed at a privileged tax rate and generally without consideration of any other income. After the payout, wealth taxes apply annually, but they are relatively low. Occupational pensions are, in principle, contribution funded. However, the pensions of many retired persons exceed their savings, which leads to a redistribution of wealth from the active to the retired population.

The third pillar consists of voluntary private contributions. Conditional on employment, individuals older than 25 years can deduct their contributions from taxable income up to a yearly amount of 6739 CHF (in 2014); a higher threshold applies to self-employed individuals. As occupational pensions, third-pillar accounts can be withdrawn before retirement for the purchase of a property or the formation of a business. Withdrawal is possible up to 5 years before ordinary retirement age.

Table 1 gives an overview of mean pensions and coverage rates of the Swiss pension. Because there are no official statistics on coverage rates and average pensions in the second and third pillar, I rely on the SILC data from 2015. The statistics underlines the importance of occupational pensions in Switzerland in terms of both payouts and income after retirement.

**Table 1:** Overview of old-age benefits in Switzerland

Pension scheme	Mean gross pension (CHF/ month)^1^ 2014	Share of recipients 2014^2^	Mean pay-out at retirement (CHF) 2015^3^
OASI	1883	98.8%	
Occupational pensions	2577	49.0%	167,805
Third pillar		55.2%	59,028

^1^Only individuals receiving a pension. Source: own calculations from CH-SILC 2015

^2^OASI and occupational pensions: shares among retired individuals living in Switzerland. Third pillar: share of individuals aged 51–60 years. Source: own calculations from CH-SILC 2015

^3^Source: New pension statistics (Swiss Federal Statistical Office, 2019b)

**Wealth** inequality in Switzerland the high average wealth in Switzerland is well documented in the national accounts by the Swiss National Bank (SNB). In contrast, there has been little research on wealth distribution. All we know so far on wealth inequality is based on tax records. The Swiss Federal Tax Administration (2019) provides annual estimates of the Gini coefficient, which amounted to 0.86 in 2015. Dell, Piketty and Saez (2007) and Föllmi and Martínez (2017) analysed top wealth shares, which showed that the richest 1% owned 40% of all taxable wealth in 2011. This is a concentration greater than in any other country in the World Inequality Database (WID) but close to wealth concentration in the USA. One reason for the high concentration at the top of the wealth distribution is the high share of millionaires and billionaires residing in Switzerland, but another is the absence of all types of pension wealth in tax records. Other problems with tax data are that it undervalues housing wealth and refers to tax units rather than households. The most recent global wealth report (Shorrocks, Davies and Lluberas, 2018), as well as an analysis of survey data (Ravazzini, Kuhn, Brulé and Suter, 2019), estimates the Gini-coefficient of Switzerland to be 0.74 and 0.76 respectively, which is around the worldwide average and rather high in European comparison.

## Data

The main database of this study is the SILC survey from 2015 conducted by the Swiss Federal Statistical Office (SFSO). This survey contained an experimental wealth module with separate questions on financial, real estate and third pillar assets, as well as mortgages and valuables for 7468 households and 10,164 individuals. Missing values have been imputed by the SFSO, and survey weights are used to correct bias in terms of age, sex, income and other characteristics. Although the survey is a good basis for estimating net worth for the general population, some limitations of the database need to be mentioned. First, the survey data do not cover the top of the wealth distribution well. Considering the highly skewed distribution of wealth, tax records are better suited for this purpose. For this reason, I do not provide estimates for the top shares of the wealth distribution. Second, information on private loans (apart from mortgages) and businesses is lacking. Third, the value of real estate does not always refer to the current market value, but rather to taxable values of houses (16% of households), purchase price (17%) or insurance values (6%). To correct this bias, all values have been converted to current market values using the region-specific real estate price index of the SNB and conversion-rates used by the Swiss National Tax Conference (see Ravazzini et al., 2019 for details).

To estimate pension wealth, the SILC data were complemented with administrative records. The different databases were linked on the basis of a project-specific contractual agreement. Most importantly, the survey data were linked to the federal income registry, which contains yearly income records as far back as 1981. As the registry contains all the income sources that contribute to statutory pensions, it is perfectly suited to simulate future pensions. In addition, several population registries were used, namely the registry containing the population at the end of 2014 (Statpop), the marriage registry, the divorce registry, the birth registry and the death registry. These records were used to find marriage dates to apply income splitting during marriage and birth years of children used to estimate credits for child rearing.

Most of the administrative records could be merged with unique anonymised social security numbers. Probabilistic linkages using birth, marriage, divorce or bereavement dates have been applied for events that occurred before 2010 where a social security number was not available (see Additional file 1 for details). The matching rate with the income registry amounts to 99.5% for individuals in the age range between 21 and 65. Marriage dates could be identified for 98.8% of married individuals. The linkages necessary to obtain the birth years of children were more complex, as several data sources were involved and probabilistic linkage was necessary in a few cases. Because the true number of children is unknown, there are no matching rates to be reported. Of all the children that are listed in the SILC data (children living with their parents), 97.3% could also be found in the population registries. In particular, grown-up children who were born outside of Switzerland could be missing. Two reasons explain the high matching rates for data linkage: social security number was contained in the sampling frame and the linkage required no additional consent from survey respondents.

## Estimation of pension wealth

### The present value approach

The estimation of pension wealth involves many assumptions about the future in terms of personal earnings, legislation and characteristics of the pension scheme. I follow the present value approach proposed in the OECD guidelines (2013). Although it relies on fewer assumptions than alternative methods, assumptions on risk adjustment, discount rates, borrowing constraints and future policy changes are necessary (Davies & Shorrocks, 2000).

The present value of entitlements from a pension scheme *p* (statutory or occupational pension) in year *t* is,

1 $$ {\mathrm{PV}}^p={\sum}_{t=0}^{T-a}{s}_{a,t}\times \frac{1}{{\left(1+i\right)}^t}\times {\mathrm{pension}}_t^p $$

with *s*_*a*, *t*_ denoting the probability of a person of age *a* surviving until year t, *T* − *a* indicating the remaining maximum lifespan differentiated by sex and birth cohort and *i* representing a constant discount factor (here 2%). I do not take inflation explicitly into account and assume that pensions will increase at the same rate as inflation. This avoids assumptions on future inflation rates and wage progression, which would be necessary to estimate pension streams.^2^ A retired person receives the pension from period *t* = 0 (here 2015) onward. A non-retired person receives the pension starting in a future period *t* > 0, when the person reaches regular retirement age. If survivor benefits are provided, these also have to be included in the possible stream of payments, with the appropriate probabilities attached. For the application in Switzerland, I only consider entitlement from survivors’ pensions if such a pension has already been received. To assess the potential bias of this omission, I simulated survivors’ pensions as a sensitivity analysis. Simulations with alternative discount rates are also presented as a sensitivity analysis.

If individuals receive a pension, the measurement of pension wealth is relatively straightforward. Following the accrual method from Eq. (1), each annuity is weighted by the probability of being paid (survival probabilities published by the SFSO, 2019a) and discounted to the present value.

The estimation of individuals’ entitlements before retirement is more complex as no pension can be observed. In a defined contribution scheme, the current value equals the equity accumulated in the fund so far and not yet withdrawn or rolled over into an annuity. In a defined benefit scheme, the present value is the pension benefit that individuals would receive from the current scheme at retirement time assuming that the persons would not earn additional benefits in the future. This means that I assume that the persons will not be employed between the current time and the time of retirement. For applying Eq. (1) to estimate the pension stream, each payment needs to be converted to its present value and weighted by the survival probability.

### Pension entitlements for retired persons in Switzerland

The estimation of pension entitlements is relatively straightforward for retired individuals, as ongoing pensions are recorded in the SILC data. Nevertheless, the estimation process involves several assumptions and coding decisions that need to be mentioned.

In some cases, pensions measured in 2014 are bad predictors for future pensions and need to be adapted. For the first pillar, this is the case for irregularly high pension amounts, for individuals who received a pension during only part of 2014 and for married individuals whose partner is not yet retired. For the second pillar, adaptations are required for individuals who received the pension only during part of 2014. Moreover, the plausibility of high pension amounts was checked. An additional documentation describes this in more detail (see Additional file 2).

**Fig. 1:**
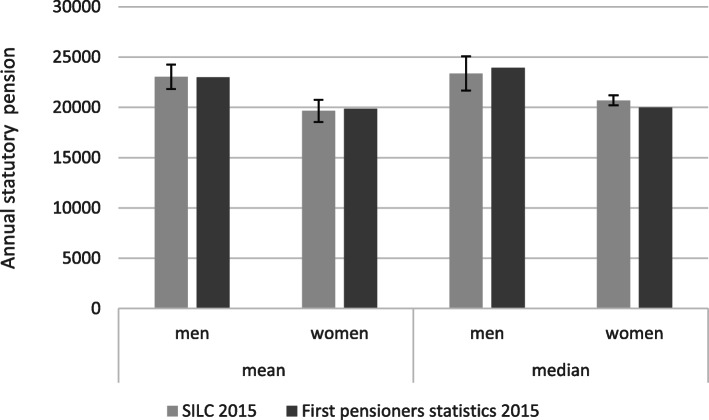
**Accuracy of first pillar pension wealth of retired individuals** Note: SILC sample: individuals who reached regular retirement age in 2014 (*n* = 125 women, 101 men). Widower pension excluded, survey weights applied. New pension statistics: individuals before or at regular retirement age who received their first pension in 2015 (*n* = 40,966 men; 44,116 women), weighted average. Source: CH-SILC 2015 (own calculation) and Swiss Federal Statistical Office (2019b)

The data quality of statutory pensions in the SILC data is very high because variables rely on information from the pension registry (Swiss Federal Statistical Office, 2017b). The critical cases for data quality are individuals who transition to retirement because their pensions needed adaptations. Figure 1 compares mean and median values of the statutory pensions in the SILC data with the new pension statistics for individuals at ordinary retirement age or younger. Values of the two data sources are very close (0 to 4% difference) and within the 95% confidence interval of the SILC data.

**Fig. 2:**
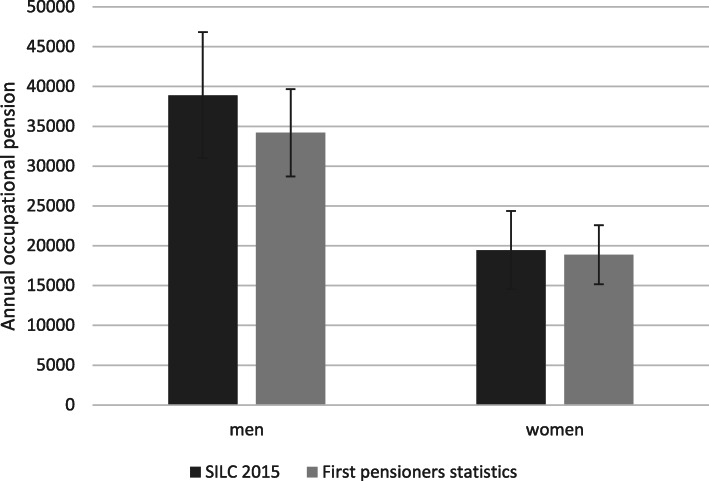
**Accuracy of second pillar pension wealth of retired individuals** Note: mean values. SILC sample: individuals who reached regular retirement age in 2014 and received an occupational pension, survey weights applied. New pension statistics: individuals before or at regular retirement age who received their first pension in 2015 (*n* = 22,543 men; 13,207 women), weighted average. Source: CH-SILC 2015 (own calculation) and SFSO (2019b)

Figure 2 compares entitlements for occupational pensions of retired individuals. For women, the mean in the new pension statistics differs only by 3% from the mean in the SILC data. For men, estimates are 13% higher than in the SILC data but remain within the 95% confidence interval. Some very small pensions might not have been declared in the SILC survey but be captured in the new pension statistics.

### Pension entitlements of non-retired persons in Switzerland

Entitlements of non-retired individuals are computed on the basis of their earning history. The accrual method assumes that the current legislation will remain unchanged until regular retirement age. For the statutory OASI pensions, the simulation takes account of the income history, credits for child rearing and equal splitting of income earned during marriage between spouses. Because pensions are computed based on the same administrative records that are used to determine the actual pension level, the estimation should be rather precise. Nevertheless, approximations were necessary in some cases. Earnings before 1981 are not recorded. Therefore, imputations for missing earnings were performed for women born between 1951 and 1959 and men born between 1950 and 1959. Older persons who already reached ordinary retirement age by 2014 usually receive a pension. For younger persons, earnings before 1981 are not relevant for the pension level. Another problem arises for divorced individuals because information of the ex-spouse’s income history is lacking, and income splitting for the years of marriage cannot be applied.^3^ Similarly, information on any previous marriages of those who remarried after a divorce is lacking. Finally, credits for child rearing might be underestimated if not all children were identified in the administrative records.

**Fig. 3:**
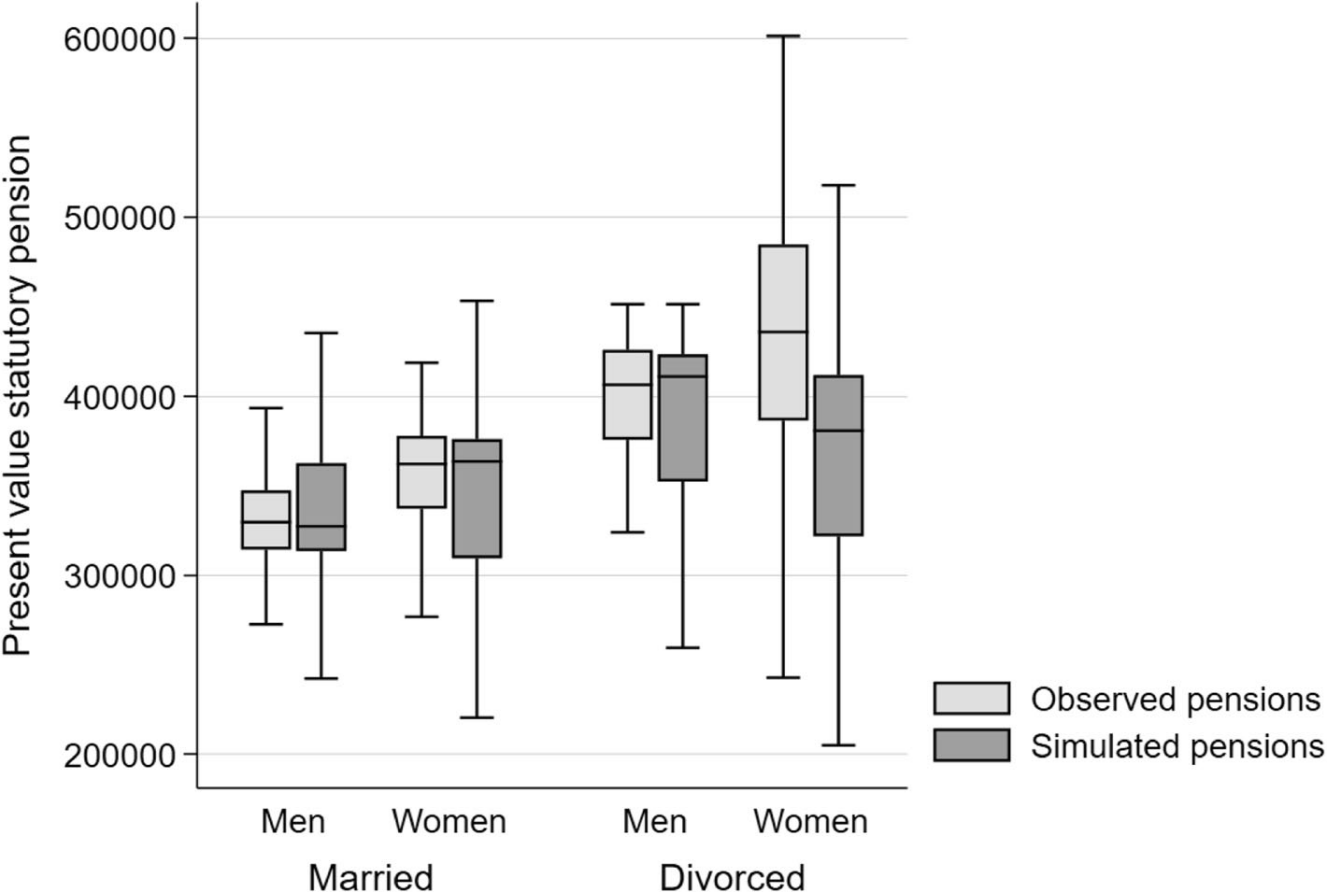
**Accuracy of simulated statutory pension wealth for non-retired individuals by civil status** Note: individuals who reached ordinary retirement age between 2012 and 2014 (*n* = 283 men, 298 women). Source: linked data from SILC 2015 and administrative records, own calculation

To test the quality of the present value of non-retired individuals, I compare observed pensions with simulated pensions for the same individuals. This is possible for people who have retired recently.^4^ Figure 3 compares the entitlements from observed and simulated pensions for individuals who reached official retirement age between 2012 and 2014 and who receive a statutory old-age pension. The figure shows a downward bias in pension wealth for divorced women because income splitting during marriage years was ignored due to data restrictions. Moreover, women have higher entitlements than men, due to their higher life expectancy. Although the estimation of the first pillar entitlements can be considered as accurate, the simulated pensions should however not be used to study the impact of divorce on pension wealth.

I turn next to entitlements for occupational pensions for non-retired individuals. The present value equals the assets that have been accumulated so far. Therefore, it is not necessary to estimate the pension stream. Nevertheless, many assumptions are required. I assume that the entire income above the minimum legal threshold contributes to the second pillar (up to the legal value of 846,000 CHF per year). To compute the insured income for men, I subtract the entire coordination deduction, which defines the lower level from which onward earnings contribute to occupational pensions (between 16,590 and 25,320 CHF depending on the year) from the employment income. For women, I subtract only half of the coordination deduction. The reason for the different treatment is that many pension schemes apply a reduced coordination deduction for part-time workers. Most women in Switzerland, but very few men, work part time. Unfortunately, the hours worked in the past that would allow for more fine-tuned scenarios are not available.^5^ For the contribution rates, I assume an average rate of 18.27 in 2015 (Federal Social Insurance Office, 2017, p. 28) and take into account differences between age groups and economic sectors (see additional file 2 for details). For the interest rates, I assume the minimum interest rate defined by the federal council for each year.

Additional (voluntary) contributions to the second pillar as well as premature withdrawals could not be considered in these estimates.^6^ By ignoring purchases into and withdrawals from the pension fund, the inequality of occupational pension wealth might be underestimated. Firstly, only individuals with a positive net worth are able to transfer assets to the pension fund. Secondly, a transfer of financial assets into the second pillar is a means to reduce taxes. The transferred amount can be deducted for income tax in the year of the transfer and will not be subject to wealth tax as long it remains with the pension fund. Therefore, purchases are most attractive for individuals with high income subject to wealth tax. Thirdly, premature capital withdrawal is most attractive for individuals who have no access to other assets.

**Fig. 4:**
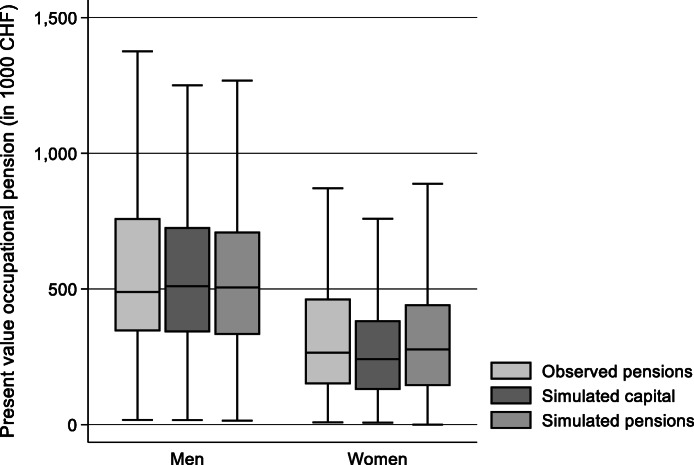
**Accuracy of simulated second pillar pension wealth for non-retired individuals** Sample: individuals aged between 58 years and regular retirement age who receive an occupational pension of at least 6000 CHF per year (*n* = 611). Widowers have been excluded. Source: linked data from SILC 2015 and administrative records (own calculations)

To test the quality of the estimate, I compare again the simulations with observed pensions for the same sample of individuals who transitioned recently into retirement. Figure 4 shows the present value from observed pensions, from the simulated pensions and from the simulated capital. The mean values of the simulations are slightly lower than the mean present value from the observed pension stream. For simulated capital, the difference amounts 2% for men and to 18% for women. This difference for women is reduced to 6% when assets are transformed to annuities assuming a conversion rate of 6.8%. Women’s higher life expectancy explains again the difference between the present value for the pension stream and the accumulated capital. Despite this difference, I will rely on the current value of the capital stock for further analysis as it does not require assumptions on conversation rates for annuities, discount rates and life expectancy. Another problem of this comparison is that some individuals might have withdrawn part of their entitlements as a lump sum, which leads to lower annuities.

Finally, I compare the estimated pension entitlements with statistics from the National Accounts, where the reported claims against insurance companies and pension funds in 2014 amounted to 112,741 CHF per capita. Per capita wealth from occupational pensions in the SILC data is 123,264 CHF, which is 9% higher. It is impossible to know to what extent this difference is due to the partial underfunding of retired individuals’ pensions and to what extent due to an inaccurate simulation. Therefore, and because of the conceptual differences between the SNA and the present value approach, I do not adjust the entitlements of occupational pensions to fit the national accounts.

## Results

### Individual pension wealth

**Fig. 5:**
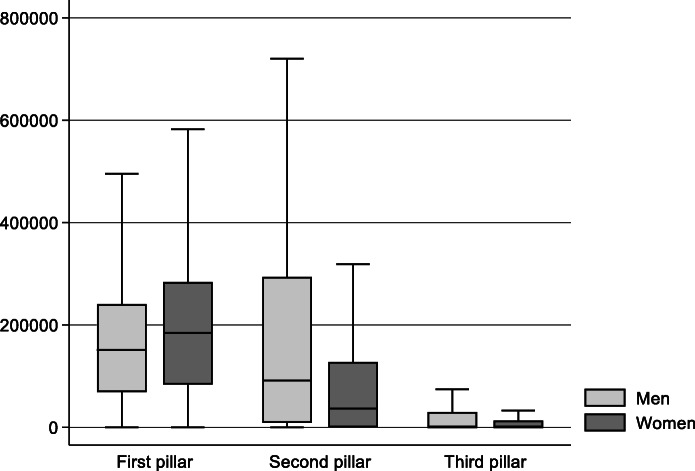
**Individual pension entitlements in 2015: mean values by sex** Notes: individuals aged 18 years and older (*n* = 13,853); own calculations; survey weights applied. Source: linked data from SILC 2015 (experimental data for wealth from 7 June 2018) and administrative records

I will first discuss adults’ pension entitlements for the three pillars. Figure 5 depicts the distribution for men and women and confirms that pension entitlements are important wealth components. With a mean value of 165,000 CHF for men and 195,000 CHF for women, the statutory pension (first pillar) is the most relevant pension asset. Women’s entitlements exceed men’s despite their lower lifetime income. The first explanation is women’s higher life expectancy. Indeed, men’s pensions are slightly higher when we look at monthly pensions from the OASI. Further reasons are income splitting during years of marriage that redistribute entitlements in favour of the women, women’s lower retirement age and the weak relationship between earnings and pension levels in the first pillar. In contrast to the first pillar, gender differences are large in the second and third pillars. Men’s entitlements for occupational pensions are more than twice as high as women’s (203,000 CHF versus 100,000 CHF, respectively), reflecting men’s higher lifetime income. Moreover, women who work part-time are more strongly affected by the coordination deduction of around 25,000 (which has varied over the years) for contributive income. Women’s higher life expectancy has only a small influence on entitlements for the second pillar because the present value of non-retired individuals refers to the accumulated capital instead of a pension stream. The third pillar entitlements are much lower in comparison to other pension wealth. With a mean value of 23,000 CHF for men and 11,000 CHF for women, gender differences are large and reflect differences in lifetime earnings. The low amounts can also be explained by the fact that individuals need to withdraw their third pillar by 70 years of age at the latest.

Inequality levels differ strongly among the different pillars of the Swiss pension system. With a Gini-index of 0.36, inequality is lowest for the first pillar. This reflects the strong redistributive component of the OASI. Inequality is considerably higher in the second pillar with a Gini-coefficient of 0.69. Many individuals have a value of zero, and pension levels vary strongly among those covered by pension funds. Accordingly, the median value is much lower than the average value (55,300 CHF versus 151,000, respectively), and the maximum present value with 4.7 million is rather high. As expected, inequality in the occupational pensions exceeds inequality in employment income. The third pillar shows the highest inequality level (Gini-coefficient of 0.82) because only 37% of adults hold a third pillar account.

**Fig. 6:**
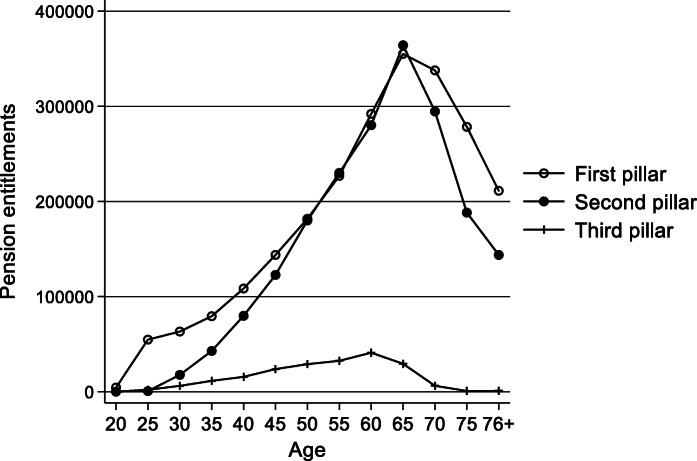
**Personal pension entitlements in 2015 by age** Note: values refer to the mean values of 5-year age groups. Weighted data. Source: linked data from SILC 2015 (experimental wealth data from 7 June 2018) and administrative records

An important part of inequality in pension wealth can be explained by age. By construction, pension entitlements accumulate over the life course until retirement age. The trends shown in Fig. 6 reflect the legislation behind the three pillars. Accumulation starts at the age of 20 for the OASI and at the age of 25 for the occupational pension wealth and the third pillar. Entitlements decline after retirement in line with the remaining life span. Third pillar assets need to be withdrawn by the age of 70. Inequality in pension entitlements is therefore largely caused by legislation and the simple fact that pensions stop with the end of life.

Because young individuals are likely to experience upward wealth mobility, inequality between age groups and within age groups has different implications. To distinguish the two, I decomposed the Theil index by eight age groups, which is shown in Table 2. Whereas age explains 73% of the inequality in statutory pensions, age makes up a smaller part of the inequality in occupational pensions (34%). Finally, age groups are least relevant for the third pillar, as they explain only 22% of total inequality. If we look at inequality within age groups only, the Theil index of occupational pensions is more than 10 times higher than the Theil index of statutory pensions (Table 2). This demonstrates the strong redistributive component of the OASI. With a Theil-index of 1.2 for within inequality, inequality in third pillar entitlements remains high even after isolating the age effect. Again, this can be explained by the large share of individuals who do not hold a third pillar account. Considering total pension wealth, inequality amounts to a Theil-index of 0.38, which is slightly higher than that for the first pillar (0.22). The third pillar entitlements have only a small influence on the inequality of total pension wealth because average levels are rather low compared to statutory and occupational pension wealth. More than half of the inequality can be attributed to age groups. Therefore, age has a large impact on pension wealth but inequality remains rather high in the second and third pillar even after singling out differences between age groups. In particular, inequality in pension wealth is very high for the elderly.

**Table 2:** Decomposition of individual pension wealth in 2015 by age groups

	Pop. share	First pillar		Second pillar		Third pillar		Pension wealth	
		Theil	Contr.	Theil	Contr.	Theil	Contr.	Theil	Contr.
18–20	4%	1.66	4.0%	0	0	0	0	1.59	1.1%
21–30	16%	.05	1.3%	1.09	1.2%	1.46	3.7%	.10	0.1%
31–40	17%	.07	2.8%	.44	3.3%	1.03	8.8%	.15	3.1%
41–50	20%	.06	5.0%	.44	10.0%	0.92	18.4%	.17	8.6%
51–60	17%	.05	5.5%	.52	16.6%	1.14	26.6%	.18	12.6%
61–65	7%	.04	2.3%	.54	10.6%	1.67	13.4%	.16	6.5%
66–75	11%	.04	3.2%	.78	16.0%	3.43	5.7%	.16	7.9%
76 +	8%	.03	1.5%	1.10	8.3%	3.56	1.1%	.16	3.5%
Within		.06	27.3%	.59	66.1%	1.21	77.9%	.17	44.7%
Between		.16	72.7%	.30	33.9%	.35	22.1%	.21	55.3%
Total		.22	100.0%	.90	100.0%	1.55	100.0%	.38	100.0%

Note: sample size: *n* = 13,881 adults. Source: linked data from SILC 2015 (experimental wealth data from 7 June 2018) and administrative records

### Augmented wealth

To address augmented wealth, I switch from the individual to the household level. This is necessary because SILC measures real and financial assets at the household level; third pillar accounts present an exception. In line with standard definitions, I attribute third pillar accounts to net worth. Augmented wealth in Switzerland amounts to 535,400 CHF per capita for the mean and to 307,700 for the median, which is very high in international comparison. Half of the augmented wealth consists of pension wealth (entitlements for the first and second pillar). The average entitlement for the first pillar is 147,000 CHF, which is slightly higher than the average entitlement for second pillar, which amounts to 123,300 CHF. Given that Switzerland already has the highest level of net worth in international comparison (Shorrocks et al., 2018), the high level of pension wealth counters the substitution hypothesis that states that private savings are low if pension wealth is high.

My estimates reveal a positive correlation between net worth and pension wealth. For the total population, the correlation coefficient amounts to 0.19. The relationship is stronger for the working age population (coefficient of 0.26) than for the retired (coefficient of 0.08). The weak correlation found among the retired can be explained by lump-sum withdrawals of second pillar entitlements at retirement and might be driven by tax incentives.

Table 3 gives further insights on the wealth portfolio by deciles of net worth (net housing wealth, bank accounts, stock and bonds and valuables). For the lower deciles, pension wealth makes up almost the entire augmented wealth. At the same time, most pension wealth for the second and third pillars is concentrated in the upper part of the distribution. The 30% richest households in terms of net worth own 60% of private pension wealth and 55% of occupational pension wealth. The highest decile stands out, as pension wealth presents only a relatively small part of their assets. This analysis refers to per capita household wealth, but findings for total household wealth are shown in an additional table (see Additional file 3).

**Table 3:** Distribution of per capita household wealth in 2015 by net worth decile

	Net housing and financial wealth		Third pillar		Occupational pensions		Statutory pensions		Augmented wealth
1. Decile	− 7756	− 5%	1002	1%	111,759	71%	51,876	33%	156,880
2. Decile	3146	2%	1273	1%	103,780	66%	48,111	31%	156,309
3. Decile	10,132	6%	4145	2%	105,375	60%	56,860	32%	176,511
4. Decile	23,063	11%	7301	3%	114,380	53%	71,135	33%	215,879
5. Decile	49,002	19%	10,754	4%	118,934	45%	85,034	32%	263,724
6. Decile	89,976	26%	13,924	4%	133,192	38%	111,249	32%	348,340
7. Decile	147,837	33%	16,551	4%	150,145	34%	133,320	30%	447,854
8. Decile	234,899	40%	21,144	4%	174,439	29%	164,176	28%	594,659
9. Decile	411,390	46%	27,291	3%	221,310	25%	228,516	26%	888,507
10. Decile	1,553,209	74%	35,232	2%	236,336	11%	282,598	13%	2,107,375

Note: Decile of net wealth. Housing and financial wealth includes real estate, bank accounts, stocks and bonds and valuables. Per capita values include all household members. Percentages refer to the percentage of augmented wealth

Regarding the inequality of aggregate wealth measure, which is shown in Table 4, the first two pillars of the pension system contribute to a reduction in wealth inequality. 7 Adding only the second pillar entitlements to net worth, the Gini coefficient declines by 10 points or 13% from 0.75 to 0.65.^8^ Given that entitlements from occupational pensions might be overestimated compared with the SNA and considering that voluntary transfers to the second pillar are ignored, the reduction in inequality might even be weaker in reality. The equalising effect is much stronger for statutory pensions. When added to net worth, inequality is reduced by 17 points or 23% to 0.57. Together, pension wealth of the first and second pillars reduces wealth inequality by 26% to 0.55. In both pillars, the inequality reduction presents an upper value, as heterogeneous life expectancy is not considered here due to lack of appropriate data. For occupational pensions, this bias is weaker as the present value of individuals before retirement (capital stock) is independent from life expectancy.

**Table 4:** Descriptive statistics of pension wealth and augmented wealth in 2015 (household level, values per capita)

	Mean	p25	p50	p90	Gini
Net worth	265,181 (8796)	14,325	79,333	608,865	.75 (.007)
Statutory pensions	146,953 (817)	62,045	105,486	311,846	.39 (.002)
Occupational pensions	123,264 (1324)	19,933	60,770	319,050	.61 (.003)
Augmented wealth	535,397 (9360)	138,050	307,728	1,147,761	.55 (.008)
Net worth + PV1	412,134 (8959)	100,423	223,953	852,562	.57 (.004)
Net worth + PV2	388,323 (9137)	55,593	95,495	88,398	.65 (.005)

Note: sample size *n* = 7468 households. Standard errors in parenthesis. Per capita values include all household members. Source: linked data from SILC 2015 (experimental wealth data from 7 June 2018) and administrative records

To look at the contribution of the different wealth components to wealth inequality, a decomposition analysis is required. According to a factor decomposition of the Gini index (Rao, 1969)^9^, entitlements for statutory pensions amount to 28% of net worth but account only for 16% of the inequality. Occupational pensions amount to 23% of augmented wealth and contribute 21% to inequality. Net worth constitutes 49% of augmented wealth but contributes 63% to inequality of augmented wealth. This underlines again the strong redistributive effect of the OASI in Switzerland.

To situate the results in an international context, results can be compared with those of Bönke, Grabka, Schröder and Wolff (2019) on Germany and the USA. Average wealth in Switzerland is much higher than in these countries. The gap in average wealth widens when accounting for pension wealth. The equalising effects of pension wealth in the three countries are comparable. In Germany, the Gini index decreases by 33% or 0.26 points when pension entitlements are added to net worth. This is slightly stronger than in Switzerland, where the Gini coefficient declines by 25% or 0.20 points. Despite the high inequality of net worth in the USA, the equalising effect of pension wealth is weaker (− 20% or − 0.18 points). Consequently, the difference in wealth inequality between the USA and the other countries does not diminish when accounting for pension wealth.

**Fig. 7:**
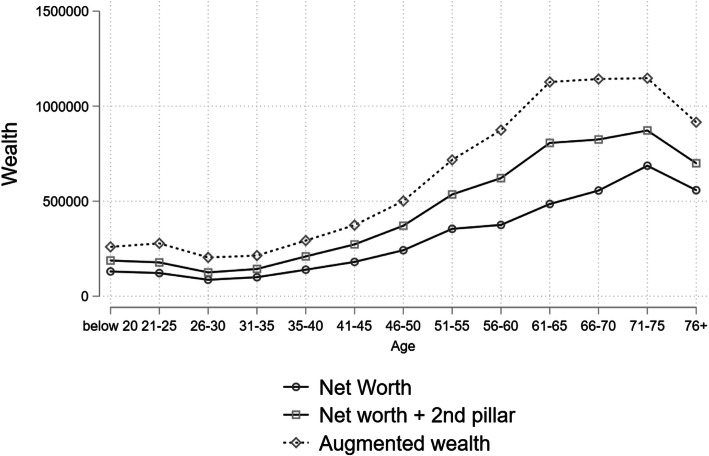
**Wealth over the life span** Source: linked data from SILC 2015 (experimental wealth data from 7 June 2018) and administrative records

Finally, Fig. 7 illustrates the accumulation of wealth over the life course. According to life-cycle theory, wealth should reach the maximum level at retirement age. However, net worth in Switzerland does not follow this pattern. Average net worth keeps increasing up to the age of 75 (cf. Fig. 7). Pension wealth explains this relationship to some extent, as lump-sum withdrawals of second and third pillar assets increase net worth around retirement age. Accordingly, pension wealth declines after retirement due to payouts and declining life span. Adding pension wealth, wealth accumulation indeed stagnates at retirement. Nevertheless, decreasing average wealth levels are only observed after 76 years. Therefore, pension wealth cannot explain this relationship entirely.

This pattern is in line with an analysis of the fiscal data for the canton of Zürich that show jumps in wealth at the end of the working life and beginning of retirement age (Moser, 2019). With a longitudinal analysis at the individual level, Moser confirms that wealth accumulation continues beyond retirement age. Therefore, the pattern cannot be attributed to higher mortality of individuals with lower wealth. However, it needs also to be noted that the SILC data include only private households. Some of the dissaving once people move to an institution is ignored in this analysis.

Inherited wealth presents another explanation for the high wealth levels seen among those between 60 and 75 years of age. Tax data from the canton of Bern confirm that probabilities of significant inheritance are highest around 60 to 65 years, followed by the age range of 66 to 75 years (Jann and Fluder, 2015).

## Sensitivity analysis: pension reforms, discount rates and survivors’ pensions

In the following, I address pension reforms and conduct simulations for additional scenarios, namely an increase of statutory retirement age, different discount rates and the inclusion of widowhood pensions.

Mainly due to the rising life expectancy, the current Swiss pension system is unsustainable and needs to be reformed by a combination of increasing retirement age, additional funding or cuts in benefits. Besides economic arguments for different reforms (Börsch-Supan, 2016; Grossmann and Strulik, 2019), political feasibility is an important element to consider, as pension reforms need to pass a referendum in Switzerland. Current reform proposals for old-age pensions involve lower conversion rates for occupational pensions, increase in retirement age and increased funding (Häusermann, Kurer and Traber, 2019). Pension retrenchment seem to be the least accepted reform element by the population (Häusermann et al., 2019, p. 1074).

These possible reform elements have different redistributive consequences. Lowering conversion rates for occupational pensions has no effect on augmented wealth as measured here. Additional funding is likely to have an indirect impact on wealth inequality, but this effect depends on the source of funding and would require the development of a specific model that is beyond the scope of this article. For the sensitivity analysis, I focus on increasing the retirement age, as this is both a reform element actually discussed in political debates and one with direct redistributive consequences.

A higher retirement age lowers the present value of pension wealth. With a retirement age of 68 years, the median present value of statutory pensions decreases by 22% for women and by 18% for men compared to current retirement age (65 for men and 64 for women). The unification of pension age reduces the gender gap which is in favour of women. At the individual level, the Gini coefficient for statutory pension wealth increases from 0.36 to 0.39. At the household level, the Gini index of augmented wealth increases from 0.55 to 0.57. Entitlements for occupational pensions are not affected by a higher retirement age.

Estimates on pension wealth are also sensitive to the choice of the discount rate applied to pension streams. The real discount rate for the reference scenario is set to 2% here, following studies by the OECD (2005), Bönke, Grabka, Schröder, Wolff and Zyska (2019) and Wolff (2015). Another frequent choice in micro-studies is a rate of 3% (e.g. Bönke, Grabka, Schröder, Wolff and Zyska, 2019; Maunu, 2010). The rate of 2% rate is slightly higher than the interest rates for government bond rates, which are recommended by the OECD as the basis for estimating pension wealth (OECD, 2013).

The higher the discount rate is set, the lower is statutory pension wealth and the higher is inequality of statutory pension wealth. The estimates for occupational pension wealth are less sensitive to the discount rate because they rely only to a small extent on future pension streams. The higher the discount rate, the smaller is the redistributive effect of pension wealth. Assuming a discount rate of 1% (e.g. according to risk-free interest rates), the Gini coefficient of augmented wealth amounts to 0.5. Assuming a discount rate of 5% (e.g. according to returns to investment), the Gini coefficient of augmented wealth amounts to 0.61. The additional file 5 provides estimates for alternative discount rates ranging from 1 to 5%.

The simulation has ignored the present value of widowhood pensions due to data limitations, unless individuals already receive such a pension.^10^ To get an idea of the resulting bias, I simulated the present value of survivors’ pension imposing assumptions on future marriage, birth of children, change in statutory old-age pension following bereavement, as well as benefits granted by occupational pensions, that yield rather an upper limit for these entitlements (see Additional file 4). For statutory pensions, this simulation shows that survivors’ pensions have only a minor impact on aggregate wealth. Statutory pension wealth is increased by about 5%, mainly due to higher old-age pensions for widows and widowers and the increasing likelihood of bereavement with age. As women profit more strongly from widowhood pensions, the gender gap in favour of women for first pillar wealth is increased by these pensions. However, widows’ pensions do not affect the Gini index for statutory pension wealth. Widows’ pensions play a more important role in occupational pensions. In the simulated scenario, entitlements increase by 14% and the Gini index at the individual level decreases from 0.69 to 0.63. A major reason for this reduction is a lower gender-gap (in favour of men). At the household level, the inclusion of widowhood pensions has a much weaker impact, as gender-differences play a smaller role. Considering both statutory and occupational widows’ pensions, the addition of survivors’ pensions increases per capita augmented wealth by 6% but does not affect inequality as measured by the Gini coefficient significantly. The decomposition of pension wealth by age groups is not altered once widow’s pensions are considered. In sum, widowhood pensions are relevant for the analysis of gender-pension gap but not for wealth inequality at the household level.

## Conclusions

Wealth inequality is a key aspect of economic inequality. As in other countries, wealth differences in Switzerland are much larger than income differences. However, most estimates on the wealth distribution neglect pension wealth. When pension wealth is added to net worth in Switzerland, average wealth doubles in Switzerland. Statutory pensions constitute 28% and occupational pension 23% of augmented wealth.

When focusing on augmented wealth instead of net worth, wealth inequality is considerably lower. The Gini coefficient declines by 26% to 0.55. However, there are strong differences between the different pillars of the Swiss pension system. The statutory pensions have a strong equalising effect that reflects their redistributive character. The equalising effect of occupational pensions is much weaker. Even after considering pension wealth, wealth inequality remains high and well above income inequality.

The findings do not support the hypothesis that the pension system crowds out private savings. Households with large amounts of saving tend to have higher pension entitlements. Moreover, differences between countries do not narrow once pension wealth is considered. The life-cycle model is also only partially supported by the data, as wealth increases even after retirement up to a rather high age. This suggests that many individuals save more during their active phase than used after retirement. The concentration of wealth among the retired population raises also the question whether inequality between generations and within the elderly is problematic from a societal and economic perspective.

The linkage of survey data with administrative records enabled an analysis of wealth differences by sex and age groups. In particular, 73% of the inequality of first pillar pension entitlements can be attributed to age. In contrast, occupational and private pensions show high inequality levels, even after controlling for age. For overall pension wealth, more than half of the inequality can be attributed to age groups. My analysis has also revealed an important gender-pension gap. Women’s lower earnings over the life cycle translate directly into lower entitlement for occupational pension and third pillar assets. In contrast, women have a higher present value of future social security pensions that can be explained by life expectancy and income splitting during marriage.

This analysis comes with some limitations. While the simulations for first pillar entitlements are quite precise, simulations for occupational pensions are based on many assumptions and are subject to uncertainty. Lump-sum withdrawals or additional contributions before retirement could not be taken into account. Such changes in occupational pension wealth occur frequently through capital splits at divorces. The only realistic possibility to better estimate the assets in the second pillar is to collect this information directly in the survey or through linkage with many pension funds. Another important limitation is the ignorance of heterogeneity in life expectancy. When income-specific survival rates are used, the equalising effect of pension wealth would be substantially lower than estimated in this contribution (see Haan et al., 2019 for Germany). Besides life expectancy, several other aspects not covered in this paper are likely to have distributional consequences, notably the choice between annuities and capital payout in the occupational pension and pension reforms other than increasing retirement age are important points for future research. Finally, the importance of statutory pension wealth and its redistributive effect depend on assumptions on the discount rate.

The results have several policy implications. The finding that third-pillar wealth correlates strongly with other wealth suggests that an extension of third pillar assets in Switzerland will neither improve financial security for those in need nor reduce wealth inequality. Moreover, a lower coordination deduction in the occupational pension would be a means to reduce wealth inequality both between men and women and between low and high earners. In contrast, an increase of retirement age will not strongly affect wealth inequality.

## Supplementary information

**Additional file 1.** : This file describes the data linkage between survey data and administrative registries in detail.

**Additional file 2.** This file describes the data simulation of pension entitlements in detail.

**Additional file 3.** This file provides additional analysis to the wealth deciles.

**Additional file 4.** This file provides additional analysis for widowhood pensions and its potential effect on inequality of augmented wealth.

**Additional file 5.** This file provides main results for different discount rates.

## Data Availability

The data that support the findings of this study are available from the SFSO and the Swiss compensation office but restrictions apply to the availability of these data, which were used under license for the current study, and so are not publicly available. The linked data is not available because the linked data set has to be deleted after the completion of the study. To use the data, a new request for data linkage has to be submitted to the SFSO, by mentioning this project. Syntax used to create and analyse the data set is available from the author upon request.

## Abbreviations

OASI: Old Age and Survival Insurance
SFSO: Swiss Federal Statistical Office
SILC: Survey of Income and Living Conditions
SNA: System of National Accounts
SNB: Swiss National Bank
WID: World Inequality Database

## Footnotes

1 For example, the SBB (a large pension fund) will decrease the conversion rate to 4.96% in 2020 and to 4.73% in 2022. There have been several political attempts to decrease the minimum conversion rate of the compulsory part of the pension funds, but a reduction has been declined in the referendum in 2017.

2 Statutory pensions increase with an average of expected price and wage growth. So far, statutory pensions increased faster than inflation, which means that the present value approach might underestimate statutory pension wealth. For example, pensions have increased from 2005 to 2015 by 9.3%, whereas inflation was only 2% over this time-period. For occupational pensions, there are no legal obligations to adjust pension to inflation, but there are no statistics on the extent to which pension funds compensate for inflation. As occupational pension is likely to increase slower than inflation, I am likely to overestimate occupational pension wealth. Because occupational pension wealth is mostly based on the capital stock, this bias will be small.

3 Only in rare cases that income splitting has been explicitly asked for at the time of divorce, the splitting is already implemented in the data.

4 The younger the respondents, the better the comparability between the two approaches. Older individuals have more years before 1981 with unknown income. Furthermore, the parameters to estimate pensions have varied over the years. Pensions simulated from income records apply the parameters for 2014, whereas the pensions of retired individuals have been computed with parameters for the year they received the first pension.

5 However, I tested alternative scenarios. If I do not adjust the coordination deduction for women, I strongly underestimate women’s pensions. If I apply half the coordination deduction of men, men’s second pillar entitlements are strongly overestimated.

6 Purchase into the pension funds are allowed up to the amount that would have been accumulated if the individuals had earned the current salary since the age of 25. In the future, linkage with the new pension statistics will help to correct for lump-sum withdrawals made before retirement from 2015 on.

7 The third pillar has only a small impact on inequality. When excluding third pillar accounts from net worth, the Gini coefficient rises to 0.77, meaning that the third pillar reduces wealth inequality by 2 points.

8 The top 10% shares in the SILC data drops from 60 to 45%, when occupational pensions are added. However, the top 10% shares are underestimated in the survey as top wealth holders are not well covered in the data.

9 *G* =  ∑ *s*_*k*_*C*_*k*_, where *G* refers to the Gini coefficient of total income inequality, *s*_*k *_refers to the share of income source k in total household income and *C*_*k*_ refers to the concentration coefficient of income source *k*.

10 Reasons for the omissions are lack of data and many unknown parameters for the simulation. The eligibility for a widowhood pension depends on the marital status, year of marriage, and the age of children at the time of bereavement. In the occupational pensions, pension funds have a lot of leeway in structuring widow’s pension.
