# Supplementary material for: Augmented wealth in Switzerland: the influence of pension wealth on wealth inequality
Source: Swiss J Econ Stat. 2020 Nov 5;156(1):19. doi: 10.1186/s41937-020-00063-9 (PMC7651273; doi:10.1186/s41937-020-00063-9)
Supplement: Supplementary file 1 — Additional file 1. : This file describes the data linkage between survey data and administrative registries in detail. [file 41937_2020_63_MOESM1_ESM.docx]

# Additional file 1

# Description of Data linkage SILC – administrative data

## Data linkage with the income registry

Of the 17’164 individuals in the SILC-2015 file, 79.3% could be linked to the OASI registry. There are different reasons why there is no record in the pension registry. Firstly, individuals younger than 21 are not yet subject to social security contribution. Their present value for the first and second pillar can be set to 0. Secondly, income before 1981 is not recorded in the registry. Individuals, who did not work since 1981, have no entry in the registry. This concerns mostly older individuals who retired before 1981 but also married individuals (mostly women) who are covered in the pension system through their spouse. Restricting the SILC sample to the relevant age range between 21 and 65 (the younger have no contributions, the older receive a OASI pension), 98.6% could be matched directly. Considering also matches through the spouse (8 men and 85 women), the rate of successful data linkage augments to 99.5%. The rate is slightly lower for foreigners (98.1% for women and 99.0% for men) than for Swiss citizens (99.8 for women and 99.7% for men). In absolute numbers, only 47 individuals between 21 and 65 years who did not report a first-pillar pension and could not be merged to the registry. Either these individuals did not contribute to the first pillar so far (because they missed to contribute as an inactive person, e.g. young adults or because of recent immigration) or there was a problem with the AVS number. In the former case, individuals have a true pension entitlement of zero. In the latter case, they have a positive pension entitlement. Individuals who are younger than 23 have or have migrated to Switzerland are kept in the sample, the remaining 28 individuals without first pillar entitlements were excluded from the analysis.

## Data linkage with the population registries

The SILC data have been linked to five different population registries: The Statpop registry of the population end 2014 and four registries on events (BEVNAT marriages, BEVNAT divorces, BEVNAT living births, BEVNAT death). Table A1 lists the variables used in each registry.

The linkage with the Statpop population registry is straight forward, because social security numbers are available for all individuals in SILC. 99.3% of the individuals in SILC could be merged to the Statpop population registry, leaving thus only few individuals missing. Among the individuals that could not be matched (n=114) are those who were born in 2015 (n=23) and those migrated to Switzerland in 2015 (n=18). Because the reference point of the registry was end 2014, these individuals were not includes. Matching rates are slightly lower for foreigners (86.6%) than for Swiss citizens (98.6%).

Table A1: overview of information in the population registries

| Statpop (population registry end 2014) | AVS number  Birth date  Civil status  Date of last change in civil status  AVS number spouse  AVS number mother  AVS nb father |
| --- | --- |
| BEVNAT Marriages | AVS number man (for marriages after 2011)  AVS number woman (for marriages after 2011)  Date of birth man  Date of birth woman  Marriage date  Date of birth of common children before marriage |
| BEVNAT Divorces | AVS number man (for divorces after 2011)  AVS number woman (for divorces after 2011)  Date of birth man  Date of birth woman  Marriage date  Divorce date  Birth year of underage children at divorce |
| BEVNAT Live birth | AVS number child (for births after 2011)  AVS number father (for births after 2011)  AVS number mother (for births after 2011)  Date of birth father  Date of birth mother  Date of birth child  Marriage date |
| BEVNAT Death | AVS number deceased person (for births after 2011)  AVS number partner (for births after 2011)  Date of death  Date of birth of the deceased person  Date of birth of the partner  Birth years of underage children |

Through the linkage with the Statpop population registry, the marriage date of married individuals can be directly obtained because the date of the last change in civil status refers to the most recent marriage date. Among the 8077 respondents of SILC 2015 who declared to be married, 8054 could be linked with Statpop. 98.8% of the linked married individuals were also married according to the information from the Statpop registry. Most of the divergences in civil status between the two data sources (n=73) can be explained by marriages in 2015 (n=19) or by registered partnerships (n=35).^[[1]](#footnote-1)^ Because of this good correspondence, I could find the marriage date of 98.8% of the matched sample. The additional search for marriage dates in the marriage registry yielded only 22 additional marriage dates. Thanks to this data linkage, income splitting can be applied for almost all married couples. In contrast, I cannot apply income splitting for the years of marriage for divorced individuals, as the earnings history of the ex-spouse is unknown.^[[2]](#footnote-2)^

The population registries have also been used to search for individuals’ children. Children’s birth years are required to compute educational benefits of the statutory pensions. This linkage is more complex, as there is no single registry containing the relevant information. Rather, information of different registries need to be combined to find as many children of SILC respondents as possible. Moreover, the social security number is only recorded for events that happened after 2010. Therefore, also probabilistic merge approaches have to be applied. For the marriage registry, date of birth of both the couple and marriage date can be used. For the divorce registry, date of birth of the person in SILC and divorce date can be used.^[[3]](#footnote-3)^ For the birth registry, date of birth of the parents and marriage date can be used. For the death registries, date of birth of the partner and date of event can be used. Depending on the combination, merges might not be unique (marriages where couples have the same birth dates, or merges with one birth date and marriage dates in the marriage or birth registry). Data have therefore to be cleaned to drop obvious false merges, but probabilistic merges are unavoidable when detecting children.

To find the birth years of the different children, different steps have been applied for this linkage. First, the social security number of SILC respondents can be matched to social security number of mothers and fathers in the Statpop population registry (exact merges). In this way, most children can be easily identified. However, slightly more children can be found for women (n=8903) than for men (n=7808). Because of data linkage via the social security number, we can be confident that the children found are indeed children of SILC respondents. Children who did not live in Switzerland at the end of 2014 or children whose social security number of the parents is missing cannot be detected in the registry. I therefore searched for additional children in the different registries on population movement. The second linkage to find children was made with the birth registry using the social security number of the mother and father. A few additional children could be found (138 for women, 142 for men). Third, I identified the partner of SILC respondents via the Statpop registry and then used the date of birth of both partners as identifiers for linkage with the birth registry. This and the remaining linkages are probabilistic rather than unique, because they are based on birth dates and event dates rather than the social security number. After correction for false merges (see below), 84 (for women) and 95 (for men) additional children could be found. Forth, I used the partner identifier from SILC and used again the birth dates of both partners as identifiers for linkage with the birth registry.^[[4]](#footnote-4)^ After correction of false merges, 62 (for women) and 65 (for men) additional children could be found. Fifth, I linked the SILC respondents to the birth registry using the date of birth (of the SILC respondent) and the marriage date as identifiers. This linkage indicates many possible children, as there are several individuals with the same date of birth and marriage date. I therefore only retained those children, who were assigned to both partners of a married cohabiting couple (126 additional children for women and men). Moreover, I linked the SILC data to the divorce registry (37 additional children for women, 30 additional children for men), the death registry (1 additional child for men) and the marriage registry (3 additional children for women, 5 additional children for men).^[[5]](#footnote-5)^ Finally, I added children who live in the household from the SILC survey. Women have 333 additional children, which were not detected in the population registries. Men have 398 additional children that were not detected in the population registries.

The problem with probabilistic linkage is that children of other couples with the same characteristics might therefore be wrongly added to SILC respondents. I applied a series of corrections to limit this error. First, I identified married couples in SILC who have the same birth date as another married couple in the marriage registry and did not add children of other couples with the same birth year.^[[6]](#footnote-6)^ Second I dropped children when individuals were younger than 12 (1 woman) or older than 70 (1 woman).

Because the true number of children of respondents in SILC is unknown, it is difficult to measure the quality of the merge. One way to test the quality of the registry merge for children is to compute the share of children in SILC (children who live with their parents) that could be found in the registry data. Of all children listed in SILC, 96.1% could be found in the Statpop registry only (step 1 and 2), 97.3% could be found in the various population registries. The match rate is highest for Swiss citizens born in Switzerland (99.2% in total), followed by Swiss citizens who migrated to Switzerland (98.9%), and foreigners born in Switzerland (89.1%). Matching rates are lowest for foreigners who migrated to Switzerland (84.0%). Furthermore, the rate is higher for women (97.7% children found) than for men (96.9%). Nevertheless, the match rates are very good and educational benefits can be computed with a very little bias. The bias is most important if an individual is wrongly assumed to have no children. The omission of a second or third child leads only to a small bias, as the individual will still receive an educational benefit for the first child. If persons migrated to Switzerland when their children are older than 16, missing children do not bias the educational benefit of social security pensions either.

1. The variable civil status in SILC does not distinguish marriages from registered partnerships. 26 individuals are Single in Statpop but married in SILC, 13 of these married in 2014 or 2015 (marriage date could be found in the marriage registry). Eleven individuals are divorced in Statpop but married in SILC. 5 of these individuals married in 2014 or 2015 according to the marriage registry. One individual is married in SILC and widowed in Statpop, also this person revealed to have married in 2015. [↑](#footnote-ref-1)
2. To apply income splitting for divorced individuals for the time during marriage, the AVS number of the ex-spouse needs to be identified in the population registries. These individuals need then to be matched to the AVS registry. While the technical feasibility is given, the legal feasibility of this match needs first to be assessed, as it concerns individuals that are not part of the SILC sample. [↑](#footnote-ref-2)
3. For the linkage, only the part of the Statpop registry related to SILC (where the person is SILC is the person in the registry or the partner, father or mother of another person) was available for this project. Therefore, I was not able to identify the birth year of the ex-spouse of divorced individuals. [↑](#footnote-ref-3)
4. The date of birth of the partner can be obtained through linkage with the Statpop registry [↑](#footnote-ref-4)
5. To link these files, several preliminary merges had to be made. For divorced individuals (n=1184 in SILC), 204 could be merged via the social security number, 666 could be merged via birth date and date of divorce, 1 could be merged with the social security number of the divorced partner, and 9 could be merged using birth dates of both partners. Because not the entire Statpop registry was available for this project (only SILC respondents, partners of SILC respondents and children of SILC respondents), the possibilities for linkage with the divorce registry were limited to the search for children because no earnings history of the partner is available. Also for the marriage registry and the death registry, different approaches for the merge have been used. [↑](#footnote-ref-5)
6. 74 SILC respondents had a multiple merge with the marriage registry using the birth years of both partners for the merge. Through the variable “last change in marital status“ from the Statpop registry, it was possible to identify the right marriage. 63 (for women) and 61 (for men) children could be removed from this children file in this way. [↑](#footnote-ref-6)
