# Supplementary material for: Augmented wealth in Switzerland: the influence of pension wealth on wealth inequality
Source: Swiss J Econ Stat. 2020 Nov 5;156(1):19. doi: 10.1186/s41937-020-00063-9 (PMC7651273; doi:10.1186/s41937-020-00063-9)
Supplement: Supplementary file 2 — Additional file 2. This file describes the data simulation of pension entitlements in detail. [file 41937_2020_63_MOESM2_ESM.docx]

# Additional file 2

# Documentation for the simulation of pension entitlements

To estimate pension entitlements, I refer to legislation from 2014, because the SILC 2015 observes pensions received during the year 2014. Wealth questions refer to the beginning of 2015 (moment of the interview) or the end of 2014.^[[1]](#footnote-1)^ To present estimation of pension wealth, I discuss four cases separately: first pillar entitlements of retired individuals, second pillar entitlements of retired individuals, first pillar entitlements of non-retired individuals and second pillar entitlements of non-retired individuals.

## First pillar entitlements of retired individuals

The pension wealth of retired individuals is estimated at the basis of observed social security pensions in the SILC survey. Old age and widower pensions are taken into account. The variables on OASI pensions in SILC are based on the pension registry rather than from answers of survey respondents. All retired individuals should receive a social security pensions, the exceptions are those who never contributed to the first pillar because they lived abroad. Individuals may also postpone their pension beyond regular retirement age. The number of individuals who reached regular retirement age and did not receive AVS pension in 2014 amounts to 32.^[[2]](#footnote-2)^

Several coding decisions and adaptations to the observed pensions are necessary. Pension streams should take account of future changes in monthly pensions, if the amount received in 2014 is a bad predictor of future pension. Therefore, I made adjustments for irregularly high pensions, transitions to retirement in 2014 and retirement of the spouse. In contrast, I did not adjust for unpredictable changes, such as death of the spouse or divorce. Neither do I adjust for future changes in the legislation. I discuss the adjustments to annual pensions for different events in turn.

Irregularly high pensions: A maximum level for the first pillar pension needs to be defined. In 2014, the maximum regular OASI pension amounted to 28,080CHF per year, but some circumstances, first pillar pension might exceed this amount. Firstly, pensions of individuals who deferred the OASI pension beyond regular retirement age amount up to 130% of the maximum pension level at regular retirement age. Secondly, individuals with low income receive means-tested supplementary pensions (Ergänzungsleistungen). Excluding unique supplements (e.g. for doctoral visits), the pension can amount to up to 37,434 per year for singles and to 53,822 for married couples.^[[3]](#footnote-3)^ Thirdly, retired individuals with dependent children receive an additional child pension of 40% of their yearly pension (maximum amount of 940 CHF per month). Supplements for children are paid until the child is 18 and concluded his or her first education. Considering these possibilities, I topcode social security pensions at 130% of the maximum level of the standardised first pillar pension (36’660 CHF per year). This is the highest possible amount for the future pension stream (in the case of postponed retirement) and close to maximum levels for dependent children and supplementary benefits insurance. There is no plausible reason, why a regular yearly pension might exceed this threshold in the longer term. This top coding affects 48 individuals (1.4% of individuals receiving a first pillar pension). I do not adjust small pensions, as such amounts are possible.

Transition to retirement: Individuals who transitioned into retirement in 2014 only received a pension during part of that year. Therefore, the future annual pension will be higher than the amount received in 2014. To estimate the yearly pension in future years, information on the number of month that the pension has been received in 2014 is required. I use information on the number of month from the pension registry to standardise the pension to 12-month. Pension levels of 190 individuals have been adjusted in this way, 179 at regular retirement age. The standardised pensions are topcoded at 28,080 CHF per year (maximal regular pension) to avoid implausibly high pensions due to error in the number of month (9 adjustments). This standardisation increased the average pension of those who reached their regular retirement age in 2014 from 11,128 to 18,926 for women and from 12,413 to 23,241 for men. To check whether this standardisation yields plausible annual pensions, I compare the estimations to pensions of older individuals, assuming that pensions do not vary strongly between adjacent birth cohorts. Figure A2_1 shows that after the standardisation, the estimated yearly pension for women born 1950 and men born 1949 is similar to the pension levels of slightly older individuals.^[[4]](#footnote-4)^ For women, younger cohorts seem to have slightly lower pensions, the opposite holds for men. This different trend can be explained with later pension adjustments when the partner retires, which I will look at more closely below.

Figure A2_1: Standardisation of first pillar pension of retired individuals


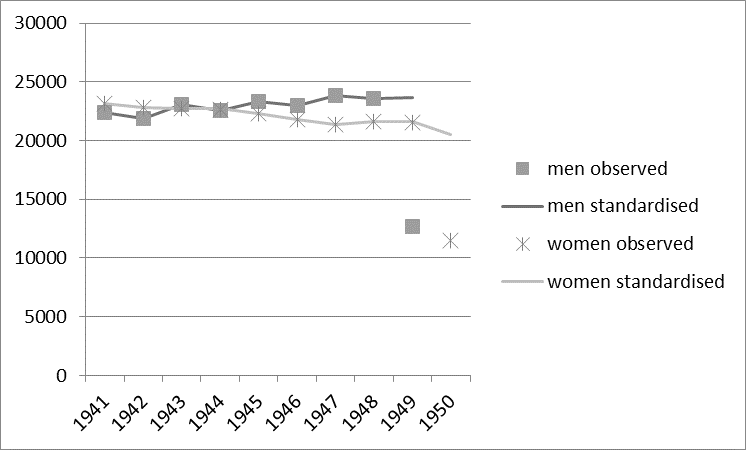


Source: CH-SILC 2015

Retirement of the spouse: A further adjustment of the reported pensions has to be made for married individuals who retire before their spouse. Once the married partner transitions to retirement, the first pillar pension is adjusted in two ways. Firstly, the maximum pensions a couple can receive is capped at 150% of the maximum individual pension. Moreover, the income earned during marriage is equally split between partners, which means that the pension is re-estimated using the new average income. Low earning individuals who have a high-earning partner, expect a pension increase. Individuals, who receive the maximum individual pension, face a cut in the pension because of the ceiling effect. Because men have higher earnings than women, the adjustment is gender-specific. For most couples, the pensions of women increase once their partner retires. In contrast, the pension of men tends to decrease once their spouse retires. If these future changes in pension in the pension stream are ignored, pension entitlement will be biased.

In SILC data 2015, 278 married individuals face a change in their pension level because their spouse is not yet retired. It is not a priory clear how this adjustment should be taken into account, as the adjustment depends on the future earnings of the non-retired spouse.^[[5]](#footnote-5)^ I therefore apply the same adjustment to all individuals concerned. To estimate the income change, I compare the average pension of married women whose husbands have already retired with the average pension of married women whose husbands have not yet retired. I find that pensions are 17% higher if the husband is retired. For men, the pension is 21% lower if the spouse is retired. I apply these adjustments to the pension stream for all years where both spouses are retired. Moreover, I top-code pensions at the maximum level for married couples for the years where both partners are retired, taking account of the sum of observed pension (for the retired partner) and simulated pension for the partner not yet retired. Figure A2_2 illustrates this adjustment, by showing the present value of the first pillar pension before and after the adaptation. For women, the adjustment has little impact on mean values, mostly because only fewer women (60 women compared with 218 men) reach regular pension age before their husband. In contrast, the correction has a considerable impact on men’s pension wealth in the first pillar. Due to income splitting and ceiling of the pension of married couples, men’s pension entitlements are lower after the correction. The present value of women’s first pillar entitlement is higher than men’s because of their higher life expectancy.

Figure A2_2: Present value of first pillar pension: correction for pension change of retired individuals


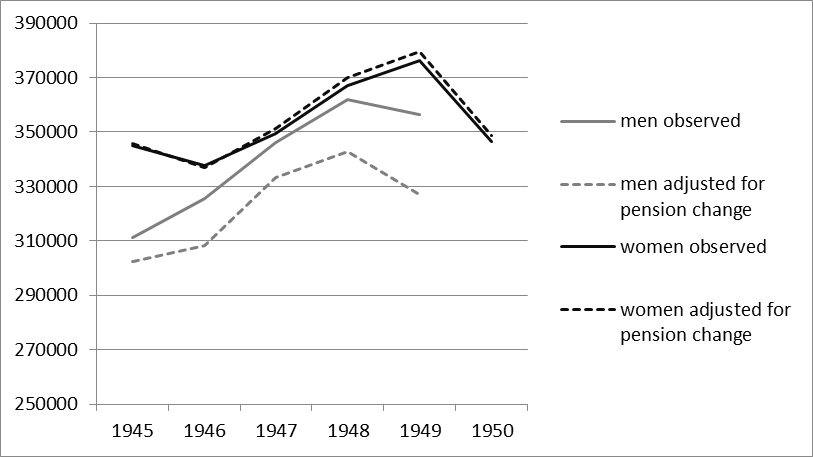


Source: CH-SILC 2015

## Second pillar entitlements of retired individuals

Similar to social security pensions, entitlements for occupational pension of retired individuals are estimated at the basis of observed pensions in the SILC survey. However, the adjustments necessary are more complicated. Firstly, there is no information from the registry on ongoing pensions, so that there might be more measurement error than for the first pillar. Secondly, there is no upper limit for pensions that allow to check the plausibility of estimates. The potential impact of outliers is also more important.

To standardise occupational pensions to yearly amounts, I use the number of month respondents said to have received the pension in 2014. Because many individuals receive their second pillar pension before ordinary retirement age (43.8% of women and 47.9% of men according to the SFSO 2019),^[[6]](#footnote-6)^ I do not use the data of birth for the standardisation.

It is important to check and edit implausibly high pension, as the bias will be multiplied when computing the pension stream. To correct potential errors, I used various auxiliary information. The occupational pension is related to lifetime income. Therefore, I compared the yearly pension with the last regular employment income (highest value between 2009 and 2014), the average employment income, and the accumulated employment income. Moreover, I looked at the number of month individuals say to have been retired in 2014. The median ratio between the yearly occupational pension and the last employment income and is 30%.

More concretely, I applied three corrections. First, I edited pensions of individuals who transitioned to retirement in 2014 (less than 12 month retired) and had regular earnings before retirement. The second pillar pension is reduced to 70% of the last salary if all of the following conditions are fulfilled (n=4): the estimated standardised pension is higher than 70’000 (1), the estimated pension is at least 80% of the mean salary since 1981 (2), the estimated pension amounts to at least 60% of the last salary (3), and the last yearly salary was higher than 30,000 CHF (4). In the second correction, I edited values of individuals where the number of month is probably not correct. I relied on the reported pension rather than the number of month if the estimated pension exceeds 75’000 and employment income in 2014 was lower than 10,000 CHF (n=3). Finally, I set values to missing if they seem implausibly high (n=28) and impute the values using the income history (see below). This is the case if the pension amounts to at least 10,000 CHF per year (1), is higher than 70% of the last regular wage (2), 80% of average earnings (3), and 3% of accumulated earnings (4) ^[[7]](#footnote-7)^, the person transitioned to retirement in 2014 (5) and is not divorced (6).

To test whether the corrections yield plausible values, Figure A2_3 shows pensions by birth cohort. The pensions of individuals in regular retirement age should be comparable to pensions of individuals who are slightly older. The mean occupational pension of individuals who reached ordinary retirement age in 2014 (women born 1950 and men born 1949) before and after the correction. After the correction, the estimated pensions are closer to pensions of older cohorts than before the correction. The highest occupational pension in SILC amounts to 252,000 CHF per year.

Figure A2_3: Old age occupational pensions by birth year

Source CH-SILC 2015, experimental wealth data from the 6.5.2018. Weighted statistics

## First pillar entitlements of non-retired individuals

According to the present value approach, I computed the pension assuming that individuals accumulate no further retirement entitlements. Future increases of the pension level (due to the inflation of consumer prices and wages) are not taken into account.

The estimation of 1^st^ pillar entitlements for individuals before retirement consists in the following step:

1. I added yearly incomes from the year the individual has turned 21 up to 2014. The incomes include all contributions in the OASI registry (employment, self-employed, contribution of inactive individuals, unemployment benefits). Women and men who were born before 1960 have some missing information in the registry, as they were older than 21 at the start of the registry. Because almost all women born before 1950 and men born before 1949 receive a first pillar pension in 2014, the population with missing entries is constituted by women born 1951-1959, men born 1950-1959 and few older men and women who have postponed their OASI pension. To impute the missing values until 1980, I compute the mean income for the years 1981 to 1985 and assume the same earnings before.
2. I split income for the years of marriage between spouses. I can do this only for couples where the earnings history of both partners is available. For divorced couples, income splitting cannot be applied and is therefore ignored.
3. I estimated supplements for education and assistance and assume that education supplements are split between parents. The amount is 7020 * age for children younger than 16. For children 16 years or older, the education benefits are 7020*16. Birth years of children are obtained from the SILC data (for children in the household) and the population registries (Statpop, BEVNAT marriages, BEVNAT birth, BEVNAT divorces, BEVNAT death).
4. I multiplied the sum of earnings and educational benefits with the upgrading factor for 2014 as indicated in the legislation. The factor depends on the year of the first OASI contribution, the maximum is 1.322 for those who already contributed to the first pillar at the age of 21. For individuals born before 1950, I assume that the contribution started at 21 or in the year of immigration to Switzerland.
5. I computed the monthly social security pension by applying the official formula. I simulated two pensions for married individuals: one for individual earnings and one with income splitting. Simulated pensions of married couples are ceiled at 150% of maximum individual pension. Although I assume no further earnings, I assumed that individuals keep contributing to the old-age security system. Almost the entire population contributes to the pension, so it is rational to assume that individuals contribute to the social security pension even if they have no employment income. Therefore, I assumed that the years between 2015 and retirement count as contribution years for the computation of pensions.
6. I computed the pension stream by multiplying estimated pension from retirement age onwards by weighting each future payment with the survival probability.
7. All future pensions are discount to current value by applying a rate of 2%.

Given that I used the same registry that will be used to compute actual pensions, the estimate should be rather precise. Figure A2_4 shows the present value of first pillar pension entitlements by birth years and sex. The estimated pension entitlements are shown both before and after income splitting of married individuals and can be compared with observed pensions. Income splitting increases the pension entitlements for women considerably and reduces pension entitlements for men. For individual who reached retirement age in 2014, the change amounts to 11% for women and to -6% for men. Without income splitting, I would strongly underestimate women’s pension wealth from the first pillar. For men, the ignorance of income splitting yields an overestimation of the present value. After income splitting, observed and estimated pension entitlements are rather close for both men and women. The difference amounts to 1% for women who reached regular retirement age in 2014, it amount to 5% for women one year older. For men who reached regular retirement age in 2014, the difference amounts to 4%, and for men who are one year older to 3%. Nevertheless, with the exception of individuals who reached retirement age in 2015, pension entitlements seem to be slightly underestimated. This gap is due to divorces, because I lack information on earnings history of the ex-spouse and could not apply income splitting.

Figure A2_4: Accuracy of first pillar pension wealth by birth year


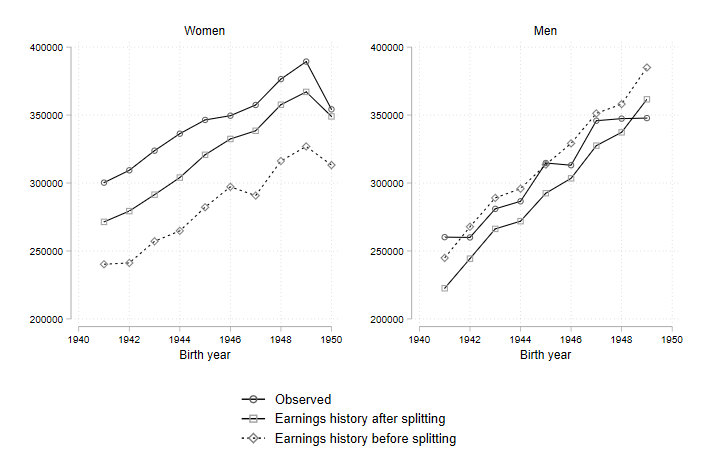


## Second pillar entitlements for individuals before retirement

There are no official statistics on contribution rates by age groups and economic sectors. To capture these differences approximately, I used the social security contributions from the SILC, as they reflect differences between age groups and economic sectors. The contribution rates are based on estimated from the Swiss Earnings Structure Surveys, which are not distributed outside of the federal administrate. From the total contribution rate, I deduct contribution for the first pillar, for unemployment insurance; income replacement order, disability insurance and sickness insurance are subtracted from that variable to estimate the contribution to the second pillar. I use the relative differences between age groups and economic sectors (multiplications) that can be applied to the average contribution rate of 18.28. I therefore assume contribution rates of 13% for 25 to 34 years, 15% for 35 to 44 years, 17% for 45 to 54 years and 19% for individuals older than 55 years. I also apply a multiplication for economic sectors (variable pl111 in SILC).

1. In the instruction for interviewers (not read to respondents), it is noted that the question refers to the estimated amount at the time of the interview or at the end of 2014. [↑](#footnote-ref-1)
2. By sex: 50% men, 50% women. By nationality: 50% do not have the Swiss nationality. [↑](#footnote-ref-2)
3. These cover the basic costs of living (19,290 CHF for singles, 28,935 for couples), health insurance premiums (depending on the canton, mean of 4944 per adult), and rents (maximum 13’200 for singles, maximum 15’000 for larger households). The first pillar pension is augmented so that individual income covers the sum of these acknowledged expenses. Further additions might be applicable for the presence of dependent children and health costs. [↑](#footnote-ref-3)
4. Widower pensions have been considered for the standardisation. [↑](#footnote-ref-4)
5. The simulated pension of the individual who is not yet retired cannot be used, because this simulated pension assumes that the partners accumulates no further entitlements. Therefore, adjusting for the present value pension would underestimate a realistic pension. [↑](#footnote-ref-5)
6. In the first pillar, only 8.3% of women and 9.4% of men received the pension before ordinary retirement age. [↑](#footnote-ref-6)
7. Assuming pension scheme with 20% of lifetime earnings in the pension fund and a conversion rate for annuities amounts to 6.8%, the annual pension would amount to 1.36% of accumulated earnings. The median value is 1.4% in the SILC sample. It is therefore unlikely that a yearly pension exceeds 3% of accumulated earnings. [↑](#footnote-ref-7)
