# Supplementary material for: Augmented wealth in Switzerland: the influence of pension wealth on wealth inequality
Source: Swiss J Econ Stat. 2020 Nov 5;156(1):19. doi: 10.1186/s41937-020-00063-9 (PMC7651273; doi:10.1186/s41937-020-00063-9)
Supplement: Supplementary file 3 — Additional file 3. This file provides additional analysis to the wealth deciles. [file 41937_2020_63_MOESM3_ESM.docx]

# Additional file 3

# Wealth deciles at the household level

Table A3_1: Distribution of household wealth by net worth decile

|  | Net housing & financial wealth | | Third pillar | | Occupational pensions | | Statutory pensions | | Augmented wealth |
| --- | --- | --- | --- | --- | --- | --- | --- | --- | --- |
| 1. Decile | -19’480 | -5% | 2’615 | 1% | 122’989 | 32% | 273’328 | 72% | 379’452 |
| 2. Decile | 8’735 | 2% | 3’015 | 1% | 116’749 | 31% | 252’677 | 66% | 381’176 |
| 3. Decile | 25’031 | 6% | 11’315 | 3% | 141’140 | 32% | 257’028 | 59% | 434’515 |
| 4. Decile | 58’559 | 11% | 18’155 | 3% | 171’966 | 32% | 284’698 | 53% | 533’378 |
| 5. Decile | 122’885 | 18% | 30’746 | 5% | 221’734 | 33% | 305’484 | 45% | 680’849 |
| 6. Decile | 246’024 | 27% | 36’316 | 4% | 288’395 | 31% | 356’662 | 38% | 927’397 |
| 7. Decile | 408’695 | 35% | 45’825 | 4% | 330’819 | 28% | 385’942 | 33% | 1’171’280 |
| 8. Decile | 614’081 | 42% | 52’853 | 4% | 379’991 | 26% | 410’959 | 28% | 1’457’883 |
| 9. Decile | 964’388 | 50% | 62’975 | 3% | 460’631 | 24% | 446’313 | 23% | 1’934’307 |
| 10. Decile | 3’249’200 | 74% | 91’716 | 2% | 573’769 | 13% | 467’058 | 11% | 4’381’743 |

Notes: Survey weights applied. Source: Linked data from SILC 2015 (experimental wealth data from 7.6.2018) and administrative records.

Note: Decile of net wealth. Housing and financial wealth includes real estate, bank accounts, stocks and bonds and valuables. Percentages refer to the percentage of augmented wealth.
