# Supplementary material for: Augmented wealth in Switzerland: the influence of pension wealth on wealth inequality
Source: Swiss J Econ Stat. 2020 Nov 5;156(1):19. doi: 10.1186/s41937-020-00063-9 (PMC7651273; doi:10.1186/s41937-020-00063-9)
Supplement: Supplementary file 4 — Additional file 4. This file provides additional analysis for widowhood pensions and its potential effect on inequality of augmented wealth. [file 41937_2020_63_MOESM4_ESM.docx]

# Additional file 4

# Survivors’ pensions

To receive a first pillar widower or widow’s pension, individuals need to be married for at least 5 years or be married and have children younger than 18 years old. In addition, women receive widow’s pension if they are older than 45 years and at least five years married at the time of bereavement or have common children irrespectively of their age. The survivors’ pension amounts to 80% of the old-age pension. After retirement, widowed persons receive either a survivors’ pension or a retirement pension, whichever is higher. The old-age pension includes a 20% supplement for widowed individuals. The same upper limits for monthly pensions apply as for old age pensions.

Due to the available data and legislation, the estimation of the survivors’ pension requires assumptions about whether and when individuals marry and whether and when they have children. I estimated the survivors’ pensions for a scenario that gives an upper limit to widowhood entitlements. For individuals before retirement, I assume that those who live with a partner marry in the following year and have children once they are 30 years old (or in the following year if they are older than 30). As survivors’ pensions are based on the deceased person’s earnings history, such an estimation can only be applied for individuals who live with a partner and for the actual partner. The survivor’s pension will have larger impact for retired individuals, as the probability of bereavement increase with age. However, I cannot simulate survivor’s pension at the basis of individual earnings, as information on the earnings history for older individuals and deceased partner is lacking. As a rough approximation, I estimated the average change in first pillar pensions following bereavement instead. Old age pensions for widowed persons are on average 28% higher than old age pension for married individuals (30% for women, 24% for men). Reasons for the rise are the ceiling of pensions of married couples (to 150% of maximum individual pensions for the couple) and supplement for widower and widows. To estimate the social security wealth for survivors’ pensions, I applied such an average increase for all married individuals in case of widowhood. The old-age pension of unmarried individuals is not adapted – I assume that lower pensions during time of marriage and higher pensions during time of widowhood cancel each other out.

For occupational pensions, I assumed that individuals receive 40% of the partner’s insured salary in 2015. If the partner is retired, I assume that the surviving spouse receives 60% of the occupational old-age pension of the deceased partner. For the simulation, I assume that individuals will receive 60% of the retired partner’s total old-age pension. This is an upper limit estimation, as the regulation only applies to the compulsory part of the old age pension. Similar to the statutory pension, I assume that all individuals living with a partner are eligible for survivor’s pension by the age of 30.

This simulation shows that mean present value of social security wealth is increased by 1700 CHF (or 1%) for survivors’ pensions before retirement age and by further 7,300 CHF (or 4%) for higher pensions after retirement (see Table A4_1). This increase is stronger for women (7%) compared to men (3%). Therefore, the gender-gap in first pillar pension wealth in favour of women is amplified by the addition of survivor’s pensions. Inequality estimates and the decomposition by age groups are not affected by statutory widowhood pensions.

Table A4_1: Present value of statutory pensions including widowhood pensions

|  |  | mean | p25 | p50 | p90 | Gini | |
| --- | --- | --- | --- | --- | --- | --- | --- |
| Adults | |  |  |  |  |  |  |
|  | Old age pension | 179’741 | 75’958 | 164’196 | 340’813 | 0.36 | [0.360-0.369] |
|  | + widowhood pensions before retirement | 181’462 | 77’840 | 167’040 | 342’815 | 0.36 | [0.355-0.364] |
|  | + widowhood before and after retirement | 188’753 | 82’205 | 174’350 | 356’716 | 0.36 | [0.353-0.362] |
| Men | |  |  |  |  |  |  |
|  | Old age pension | 164’507 | 68’560 | 150’773 | 318’667 | 0.36 | [0.356-0.369] |
|  | + widowhood pensions | 169’384 | 71’160 | 157’336 | 323’908 | 0.36 | [0.353-0.366] |
| Women | |  |  |  |  |  |  |
|  | Old age pension | 194’503 | 83’507 | 183’966 | 364’906 | 0.36 | [0.351-0.363] |
|  | + widowhood pensions | 207’520 | 93’693 | 202’419 | 384’525 | 0.35 | [0.340-0.353] |
| Household level | |  |  |  |  |  |  |
|  | Old age pension | 146’953 | 62’045 | 105’486 | 311’846 | 0.39 | [0.387-0.398] |
|  | + widowhood pensions | 154’310 | 67’267 | 114’354 | 324’835 | 0.38 | [0.379-0.389] |

Notes: Sample size: n=13,853 adults, n=6665 men; n=7188 women, n=7468 households. Survey weights applied. Source: Linked data from SILC 2015 (experimental wealth data from 7.6.2018) and administrative records

Descriptive statistics for second pillar pensions are shown in Table A4_2. The addition of survivors’ pensions increase the present value of occupational pensions by 14%. However, this increase is much stronger for women (30,000 CHF or 31%) than for men (10,600 CHF or 5%). The gender-gap in second pillar entitlements in favour of men is lowered by the inclusion of survivors’ pensions. While women’s entitlements to occupational old-age pensions are only 47% of men’s entitlements, this ratio increases to 59% once survivors’ pensions are added. In contrast to statutory pensions, survivor’s pension do affect inequality of occupational pension wealth. The Gini coefficient at the individual level decreases from 0.69 to 0.63 once simulated survivor’s pensions are added. While the declining inequality between men and women contribute to this equalising effect, the strongest reduction is observed within women's pension entitlement, where the Gini index for occupational pension wealth declines from 0.70 to 0.59. At the household level, the reduction of inequality is much weaker. The Gini index declines from 0.61 to 0.58 by adding survivors’ pensions. The decomposition by age groups does not shows a very small increase in inequality between groups (from 33.3% to 34.5%), which does not affect any of the conclusions presented in the main text.

Once we move to the household level, widowhood pensions have only a small impact on inequality. Augmented wealth increases by 6%, but the Gini index does not change significantly, when widowhood pensions are added (cf. Table A4_3). Similarly, the decomposition of inequality by age groups is hardly affected by the addition of survivors’ pensions (Table A4_3).

Table A4_2: Present value of occupational pensions including survivor’s pensions

|  |  | mean | p25 | p50 | p90 | Gini | |
| --- | --- | --- | --- | --- | --- | --- | --- |
| Adults | |  |  |  |  |  |  |
|  | Old age pension | 148’700 | 439 | 53’583 | 416’490 | 0.69 | [0.683-0.696] |
|  | & widowhood | 169’245 | 15’346 | 86’586 | 437’657 | 0.63 | [0.623-0.636] |
| Men | |  |  |  |  |  |  |
|  | Old age pension | 202’962 | 8’523 | 91’194 | 526’384 | 0.65 | [0.639-0.658] |
|  | & widowhood | 213’593 | 16’013 | 103’854 | 543’671 | 0.63 | [0.620-0.639] |
| Women | |  |  |  |  |  |  |
|  | Old age pension | 96123 | 0 | 32679 | 270430 | 0.70 | [0.696-0.713] |
|  | & widowhood | 126’274 | 14605 | 80231 | 308794 | 0.59 | [0.584-0.603] |
| Household level | |  |  |  |  |  |  |
|  | Old age pension | 121’399 | 19’439 | 59’429 | 315’245 | 0.61 | [0.604-0.621] |
|  | & widowhood pension | 138’177 | 29’626 | 75’848 | 347’913 | 0.58 | [0.572-0.589] |

Notes: Sample size: n=13,853 adults, n=6665 men; n=7188 women, n=7468 households. Survey weights applied. Source: Linked data from SILC 2015 (experimental wealth data from 7.6.2018) and administrative records

Table A4_3: augmented wealth including survivors’ pensions

|  | mean | p25 | p50 | p90 | Gini | |
| --- | --- | --- | --- | --- | --- | --- |
| Augmented wealth | 1227401 | 382855 | 824703 | 2453690 | 0.50 | [0.488-0.519] |
| & widower pension | 1298009 | 427903 | 902495 | 2567591 | 0.49 | [0.475-0.504] |

Note: widower pensions for statutory and occupational pension added. Source: Linked data from SILC 2015 (experimental wealth data from 7.6.2018) and administrative records

Table A4_4: Decomposition of inequality by age group after adding survivors’ pensions

|  | Pop. Share | First pillar | | Second pillar | | Third pillar | | Pension wealth | |
| --- | --- | --- | --- | --- | --- | --- | --- | --- | --- |
|  |  | Theil | Contr. | Theil | Contr. | Theil | Contr. | Theil | Contr. |
| 18-20 | 4% | 1.65 | 3.7% | 0.00 | 0.0% | 0 | 0.0% | 1.57 | 1.2% |
| 21-30 | 16% | 0.06 | 1.4% | 0.85 | 2.3% | 1.46 | 3.7% | 0.12 | 1.3% |
| 31-40 | 17% | 0.07 | 3.1% | 0.28 | 3.2% | 1.03 | 8.8% | 0.12 | 3.2% |
| 41-50 | 20% | 0.06 | 5.2% | 0.32 | 9.3% | 0.92 | 18.4% | 0.14 | 8.3% |
| 51-60 | 17% | 0.05 | 5.5% | 0.42 | 16.1% | 1.14 | 26.6% | 0.16 | 12.4% |
| 61-65 | 7% | 0.03 | 2.2% | 0.45 | 10.1% | 1.67 | 13.4% | 0.14 | 6.2% |
| 66-75 | 11% | 0.03 | 3.0% | 0.66 | 15.7% | 3.43 | 5.7% | 0.14 | 7.5% |
| 76+ | 8% | 0.03 | 1.4% | 0.96 | 8.5% | 3.56 | 1.1% | 0.14 | 3.3% |
| Within |  | 0.05 | 25.6% | 0.48 | 65.4% | 1.21 | 78.0% | 0.15 | 43.3% |
| Between | | 0.15 | 72.5% | 0.25 | 34.5% | 0.35 | 22.6% | 0.19 | 56.5% |
| Total |  | 0.21 | 100.0% | 0.73 | 100.0% | 1.55 | 100.0% | 0.34 | 100.0% |

Note: widower pensions for statutory and occupational pension added. Source: Linked data from SILC 2015 (experimental wealth data from 7.6.2018) and administrative records
