# Supplementary material for: Augmented wealth in Switzerland: the influence of pension wealth on wealth inequality
Source: Swiss J Econ Stat. 2020 Nov 5;156(1):19. doi: 10.1186/s41937-020-00063-9 (PMC7651273; doi:10.1186/s41937-020-00063-9)
Supplement: Supplementary file 5 — Additional file 5. This file provides main results for different discount rates. [file 41937_2020_63_MOESM5_ESM.docx]

# Additional file 5 Discount rates

The choice of the discount rates has large impact on statutory pension wealth and minor impact on occupational pension wealth. The reason is, that statutory pension wealth is based entirely on pension streams, while occupational pension wealth is only based on pension streams for retired and widowed individuals with ongoing pensions. This is illustrated in Table A5_1 for discount rates between 1 and 5 percent. At the individual level, the mean values of statutory pensions are halved (49%) compared to the standard scenario (2% discount rate) when applying a discount rate of 5%. For the second pillar, the reduction is with 8% much smaller, because only ongoing pensions are concerned. At the household level, the impact on mean values is of similar size.

The choice of the discount rate has also a large impact on inequality of statutory pensions. The Gini-coefficient estimated at the individual level ranges from 0.26 for a discount rate of 1% to 0.57 for a discount rate of 5%. The discount rate has little impact on the inequality of occupation pension wealth. The Gini-coefficient for a 1% (real) discount rate amounts to 0.69, and for a 5% discount rate to 0.67. For augmented wealth at the household level, the Gini coefficient varies between 0.50 (for 1%) and 0.61 (for 5%). The higher the discount rate, the smaller is hence the redistributive effect of pension wealth compared to net worth.

The large effect of discount rates on statutory pensions is due to the high inequality between age groups rather than within age groups. This is illustrated in Figure A5_1, which shows statutory pension wealth by age group for discount rates between 1 and 5 percent. With a discount rate of 5 percent, 85 % of inequality can be explained by age (with eight age groups, see Table A5_2), compared to 73% for a discount rate of 2 percent.

Third pillar entitlements are not affected by discount rates.

Figure A5_1: Statutory pension wealth in 2015 by discount rates


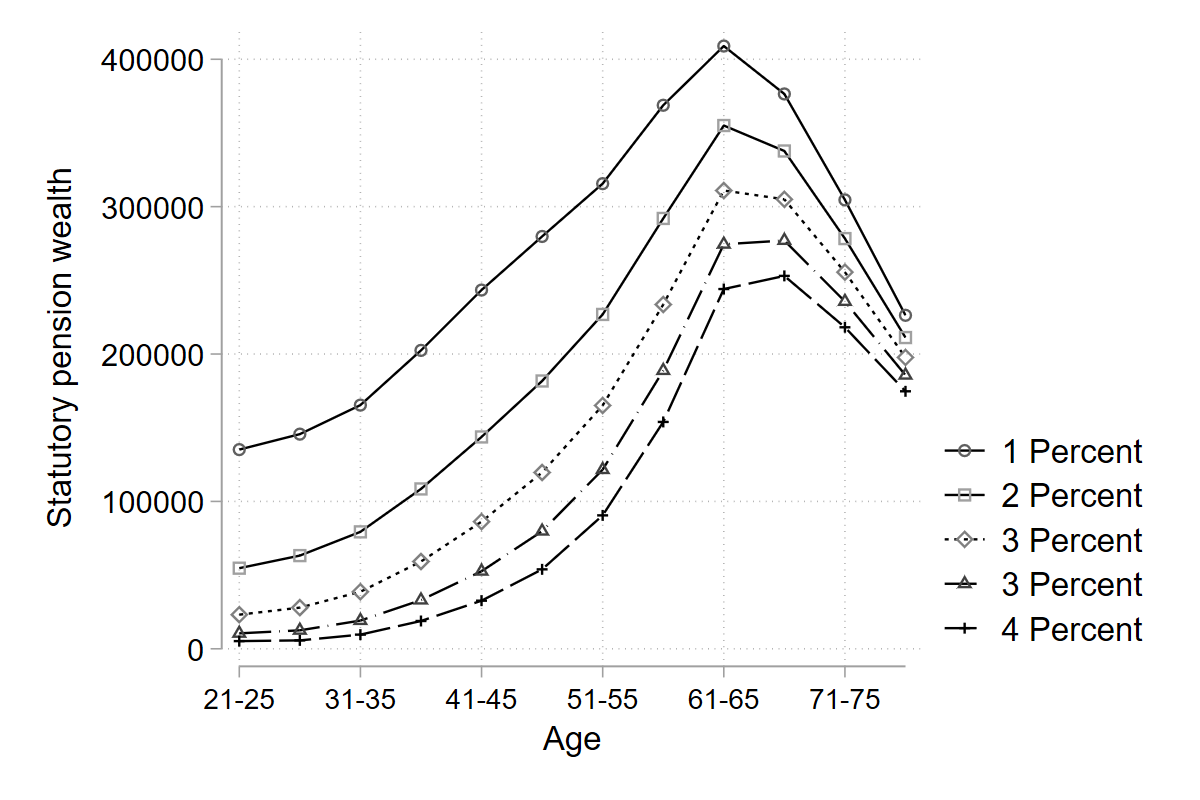


Source: Linked data from SILC 2015 (experimental wealth data from 7.6.2018) and administrative records

Table A5_1: Descriptive statistics of pension wealth and augmented wealth for different discount rates

|  |  |  |  | Mean | p25 | p50 | p90 | Gini |
| --- | --- | --- | --- | --- | --- | --- | --- | --- |
| **Individual level** | | |  |  |  |  |  |  |
|  | Discount rate 1 % | | | |  |  |  |  |
|  |  | Statutory pensions | | 251171 | 156966 | 242627 | 405538 | 0.26 (.002) |
|  |  | Occupational pensions | | 156823 | 1174 | 55641 | 435711 | .69 (.003) |
|  | Discount rate 2% | |  |  |  |  |  |  |
|  |  | Statutory pensions | | 179741 | 75958 | 164196 | 340813 | 0.36 (.002) |
|  |  | Occupational pensions | | 150984 | 1174 | 55300 | 420645 | .69 (.003) |
|  | Discount rate 3% | |  |  |  |  |  |  |
|  |  | Statutory pensions | | 137026 | 37174 | 107868 | 299123 | .45 (.003) |
|  |  | Occupational pensions | | 146043 | 1174 | 55054 | 407902 | .68 (.003) |
|  | Discount rate 4% | |  |  |  |  |  |  |
|  |  | Statutory pensions | | 109681 | 18801 | 70109 | 267039 | .52 (.003) |
|  |  | Occupational pensions | | 141826 | 1174 | 54874 | 394747 | .67 (.003) |
|  | Discount rate 5% | |  |  |  |  |  |  |
|  |  | Statutory pensions | | 91589 | 9429 | 46356 | 242759 | .57 (.003) |
|  |  | Occupational pensions | | 138198 | 1174 | 54654 | 380657 | .67 (.003) |
|  |  |  |  |  |  |  |  |  |
| **Household level (per capita)** | | | | |  |  |  |  |
|  |  | Net worth |  | 265,181 | 14,325 | 79,333 | 608,865 | .75 (.007) |
|  | Discount rate 1% | |  |  |  |  |  |  |
|  |  | Statutory pensions | | 205342 | 118660 | 173543 | 373597 | .30 (.003) |
|  |  | Occupational pensions | | 128030 | 19933 | 61310 | 332483 | .62 (.004) |
|  |  | Augmented wealth | | 598553 | 196240 | 372139 | 1220997 | .50 (.008) |
|  | Discount rate 2% | |  |  |  |  |  |  |
|  |  | Statutory pensions | | 146953 | 62045 | 105486 | 311846 | .39 (.002) |
|  |  | Occupational pensions | | 123264 | 19933 | 60770 | 319050 | .61 (.003) |
|  |  | Augmented wealth | | 535397 | 138050 | 307728 | 1147761 | .55 (.008) |
|  | Discount rate 3% | |  |  |  |  |  |  |
|  |  | Statutory pensions | | 112031 | 32801 | 68048 | 271365 | .48 (.003) |
|  |  | Occupational pensions | | 119229 | 19933 | 60402 | 308393 | .60 (.004) |
|  |  | Augmented wealth | | 496442 | 107064 | 271068 | 1096191 | .58 (.008) |
|  | Discount rate 4% | |  |  |  |  |  |  |
|  |  | Statutory pensions | | 89675 | 17718 | 44196 | 240965 | .54 (.003) |
|  |  | Occupational pensions | | 115786 | 19933 | 60280 | 296973 | .60 (.004) |
|  |  | Augmented wealth | | 470642 | 89910 | 246534 | 1056593 | .60 (.008) |
|  | Discount rate 5% | |  |  |  |  |  |  |
|  |  | Statutory pensions | | 74478 | 9891 | 29442 | 216559 | .59 (.004) |
|  |  | Occupational pensions | | 112824 | 19923 | 60002 | 289300 | .59 (.004) |
|  |  | Augmented wealth | | 452484 | 79570 | 232387 | 1017818 | .61 (.008) |

Note: Sample size n= 7,468 households, 13,853 individuals. Standard errors in parenthesis. Per capita values include all household members. Source: Linked data from SILC 2015 (experimental wealth data from 7.6.2018) and administrative records
